# Physical assessment and rehabilitation for neurogenic thoracic outlet syndrome (NTOS): A scoping review

**DOI:** 10.1177/17589983251411877

**Published:** 2026-02-05

**Authors:** Joel O’Sullivan, Christian Rushton, Marcus Bateman, Caroline Miller, Claire Stapleton, Jonathan Hill

**Affiliations:** 1 Upper Limb Clinical Specialist Physiotherapist & NIHR DCAF Fellow, School of Allied Health Professions & Pharmacy, Keele University, Keele, Staffordshire, UK & University Hospitals Birmingham NHS Foundation Trust, Queen Elizabeth Hospital Birmingham, Mindelsohn Way, Edgbaston, Birmingham, West Midlands, UK; 2 Senior MSK Physiotherapist, 1732 University Hospitals Birmingham NHS Foundation Trust, Birmingham, UK; 3 Consultant Upper Limb Physiotherapist, 1732 University Hospitals of Derby and Burton NHS Foundation Trust, Derby, UK; 4 Upper Limb Clinical Specialist Physiotherapist & NIHR Senior Clinical Research Practitioner- 1732 University Hospitals Birmingham NHS Foundation Trust, Birmingham, UK; 5 Senior Lecturer in Physiotherapy, School of Allied Health Professions & Pharmacy, 4212 Keele University, Keele, Staffordshire, UK; 6 Professor of Physiotherapy, School of Medicine, 4212 Keele University, Keele, Staffordhsire, UK

**Keywords:** NTOS, Rehabilitation, Thoracic Outlet Syndrome, Treatment, Assessment

## Abstract

**Introduction:**

Neurogenic Thoracic Outlet Syndrome (NTOS) is a complex condition that can be encountered in musculoskeletal and hand therapy services. Rehabilitation is recognised as the primary treatment for NTOS however, detail on rehabilitation components are poorly described. This scoping review aimed to identify and describe the physical assessment and rehabilitation components alongside clinical reasoning strategies that may aid therapists in the conservative management of adults with NTOS.

**Methods:**

Four databases (MEDLINE, EMBASE, CINAHL and Cochrane) were searched, utilising the PRISMA-ScR guidelines. The Template for Intervention Description and Replication (TIDieR) checklist was used to organise data regarding rehabilitation interventions for NTOS.

**Results:**

Twenty-Nine out of 1381 studies identified, met the eligibility criteria. NTOS Provocation tests (16/18 89%) were the most frequently described assessment components, followed by palpation of pertinent structures (11/18 61%) and assessment of posture (10/18 56%). ‘Decompressing the thoracic outlet’ was the main aim encountered for rehabilitation programmes. Exercise (17/19 90%) was the most frequent rehabilitation intervention identified, with stretching (*n* = 15), strengthening (*n* = 14) and neural mobility (*n* = 7) exercises being most prevalent. The Scalenes and Pectoralis muscles (*n* = 10) were the main targets for stretching whilst the Scapula (*n* = 9), Trapezius and Serratus Anterior muscles (*n* = 5) were the main targets for strengthening exercises. Other interventions identified included, posture improvement (*n* = 13), manual therapy (*n* = 10), adjuncts (*n* = 8) and activity modification (*n* = 7).

**Discussion:**

The reporting of rehabilitation techniques for NTOS is generally poor, particularly regarding treatment intensity. There is an essential need for a standardised and reproducible rehabilitation intervention for NTOS to be developed.

## Introduction

Thoracic Outlet Syndrome (TOS) is a complex clinical condition as highlighted in a Cochrane review in 2014.^1^ TOS is believed to be caused by congenital, acquired or traumatic factors, which subsequently create a compromised space for neurological (brachial plexus) and/or vascular (subclavian vein/artery) structures to pass through. Presenting symptoms vary and diagnosis is challenging due to the existence of three main subgroups (Vascular, Arterial and Neurogenic) depending on the main structures that have been compromised. Authors describe three pertinent anatomical spaces of compression in TOS: the scalene triangle, costoclavicular and subcoracoid space.^2–4^

Neurogenic Thoracic Outlet Syndrome (NTOS) accounts for up to 95% of TOS cases and is regarded as the more controversial and complex TOS subgroup due to the complexity of diagnosis and the constellation of signs and symptoms.^1,3,5^ NTOS is estimated to have a prevalence of 10 cases per 100,000 with an incidence of 2–3 cases per 100,000 per year.^6^

NTOS refers to an assumed dynamic positional compression of the brachial plexus,^7^ which can present with an array of symptoms including neck, shoulder, arm and hand pain, paraesthesia and weakness. These symptoms are often exacerbated by repetitive overhead motions and can lead to significant functional disabilities, including compromised hand function.^3,5,8,9^ Risk factors include anatomical abnormalities, female gender, previous trauma and repetitive use of upper limbs-particularly in activities such as sports that involve overhead motions. NTOS often affects individuals in the working age population.^7^ People with NTOS are known to have poor Quality of Life and high disability scores, with similar baseline physical disability scores to that of chronic heart failure and rotator cuff tear populations.^7,10–13^

Specialist rehabilitation is deemed the ‘mainstay’ of conservative management for NTOS.^9^ NTOS is often encountered and treated by therapists working in musculoskeletal (MSK) and hand therapy contexts, due to common differential diagnoses such as carpal and cubital tunnel syndrome and cervical radiculopathy^14^ and is viewed as a clinically complex condition to manage, requiring treatment by experienced therapists.^15^

A Cochrane review on the treatment of NTOS, updated in 2014, concluded that the field was dominated by low-quality evidence.^1^ Since then, progress has been made in NTOS research, including the publication of an agreed clinical diagnostic criterion (CDC)^16^ which provided a standardised framework for diagnosing NTOS. Validation of these criteria against Patient Reported Outcome Measures (PROMs) was completed in 2017.^10^ Furthermore, reporting standards have been published to encourage increased homogeneity of care through consistent reporting.^17^ However, progress in advancing the evidence base for therapy management has been slower.

### Conservative care

Currently, literature suggests the effectiveness of therapy for NTOS is suboptimal, with 60–70% of people choosing surgery due to ‘failing’ conservative care.^7,10,11^

Conservative treatments typically include rehabilitation, manual therapy, hot and cold therapy, electrophysical modalities, and the utilisation of braces and taping.^18^ A systematic review in 2011^18^ examining the effectiveness of physiotherapy treatments in NTOS, concluded they could not make a judgement about the effect of exercise on NTOS, due to the low-quality evidence.

Several narrative review articles.^18–20^ including a scoping review from 2022^21^ summarising the evidence up to 2021, have provided an overview of proposed theories and rationales for exercises included in rehabilitation programmes. However, none have described or compared programmes in detail or examined the PROMs used in NTOS studies. Furthermore, several new publications since 2022 have partially explored rehabilitation for NTOS,^22–27^ including the first randomised clinical trial which compared continued physiotherapy with surgery for persistent NTOS, which was refractory to change with initial physiotherapy.^8^

Therefore, an up-to-date synthesis of the literature is warranted, focussed on this current gap in knowledge around the content of rehabilitation programmes and the outcomes used. A scoping review is the most appropriate method to map the current therapy management of NTOS, while still maintaining a rigorous and transparent search strategy^28^ to underpin a) future consensus-based recommendations for NTOS, and b) to test the effectiveness of treatment based on such recommendations (e.g. our planned future IMPETUS (optIMal Physiotherapy for nEurogenic Thoracic oUtlet Syndrome) feasibility trial). This scoping review will lay the foundations of the IMPETUS study’s goal of developing an optimal therapy intervention for NTOS, and its findings will inform consensus-building exercises and intervention testing.

## Research aims and question

The primary aim of this scoping review was to identify and describe the physical assessment and rehabilitation components alongside any clinical reasoning strategies that may aid therapists in the conservative management of adults with NTOS. The secondary aim was to synthesise reported approaches to diagnosing NTOS and the use of PROMs.

Our over-arching research question was:

What are the key physical assessment and rehabilitation components used to manage adults with NTOS as part of conservative care?

## Methods

This review was conducted in accordance with the Joanna Briggs Institute methodology for scoping reviews^29^ and reported according to the Preferred Reporting Items for Systematic reviews and Meta-Analyses (PRISMA) extension for Scoping Reviews.^30^

### Data sources and search

MEDLINE, EMBASE, CINAHL and Cochrane databases were searched for studies published in the English language from 1st January 2000 up to 9th January 2025, using key words and MeSH headings related to the condition (neurogenic thoracic outlet syndrome) and the intervention (physiotherapy/rehabilitation). Clinical trial registries and PROSPERO were also searched. The search strategy was piloted and refined with a librarian from University Hospitals Birmingham NHS Foundation Trust, Birmingham, UK and the strategy used in MEDLINE is presented in Table 1. Searches were adapted for individual databases. Forward and backward citation tracking was also performed. The year 2000 was chosen to ensure studies reflected contemporary therapy/rehabilitation practices, and to provide an update to the systematic review conducted in 2011.^18^

**Table 1.** Search terms for MEDLINE.

Number	Search term
1	“Thoracic outlet syndrome”.ab. or “thoracic outlet syndrome”.ti.
2	NTOS.ab. or NTOS.ti.
3	Thoracic outlet syndrome
4	1 or 2 or 3
5	Physiotherap*.ab. or Physiotherap*.ti.
6	((Therap* or activit*) adj1 (physical or manual or aquatic)).ab. or ((therap* or activit*) adj1 (physical or manual or aquatic)).ti.
7	exercis*.ab. or exercis*.ti.
8	swimming.ab. or swimming.ti.
9	hydrotherap*.ab. or hydrotherap*.ti.
10	walking.ab. or walking.ti.
11	(Rehab or rehabilitation).ab. or (rehab or rehabilitation).ti.
12	Exp physical therapy modalities/
13	“Resistance Training”.ab. or “Resistance Training”.ti.
14	5 or 6 or 7 or 8 or 9 or 10 or 11 or 12 or 13
15	4 and 14
16	Assessment*.ab. or Assessment*.ti.
17	Test*.ab. or Test*.ti.
18	monitor*.ab. or monitor*.ti.
19	14 or 16 or 17 or 18
20	4 and 19

### Inclusion/exclusion criteria and study selection

The following study designs, published in English, were eligible for this review: literature reviews, systematic reviews, Cochrane reviews, primary empirical studies, treatment guidelines and clinical commentaries. Eligible participants were adults (>16 years old), with a clinical diagnosis of NTOS. This review considered studies in any context where adults (>16) could receive rehabilitation, if it was part of conservative care. This included hospital inpatient units, outpatient clinics and community settings.

Physical assessment/rehabilitation components (or proposed components) must have been described in sufficient detail to be included. Physical assessments were defined as any components of a physical examination by a clinician (e.g. joint range of motion/muscle length assessment, provocation tests) but not imaging modalities. Rehabilitation interventions were defined as any components of a rehabilitation programme (e.g. exercise, advice/education, manual therapy) but not invasive interventions such as injections or surgery. Rehabilitation interventions are often complex and multifactorial, but can be broken down into individual components, each representing a planned rehabilitation activity delivered by a trained healthcare professional.^31^ Clinical Reasoning strategies, were defined as additional pieces of information to support therapists in the decision-making process associated with managing NTOS patients, such as justification for rehabilitation components or prognostic information.^32^ (A summary of the eligibility criteria is provided in Table 2).

**Table 2.** Eligibility criteria for this systematic scoping review.

Eligibility criteria	
**Primary aim**- ‘Identify and describe the physical assessment and rehabilitation components alongside clinical reasoning strategies that may aid therapists in the management of adults with NTOS’	
**Secondary aim**- ‘To examine approaches to diagnosing NTOS and the use of PROMs’	
**Inclusion**	**Exclusion**
1. English language, full text articles published after year 1999	1. Patients with a clinical diagnosis of arterial (ATOS) or venous (VTOS)
2. Study types-	2. Treatment programme delivered as part of post-operative care only
Literature/Systematic reviews	
Primary empirical research	
Treatment guidelines	
Clinical commentaries	
3. Adults (>16)	
4. Clinical diagnosis of NTOS	
5. Physical assessment/Rehabilitation components (or proposed components)/Clinical reasoning strategies pertinent to therapy management of NTOS described sufficiently	
6. Rehabilitation delivered as part of conservative care (pre-operative)	

Using a specialist systematic review tool, Rayyan,^33^ two reviewers (JOS & CR) independently screened the titles and abstracts of all identified studies against pre-defined eligibility criteria. The full text articles for the remaining studies were retrieved and reviewed for eligibility. Any disagreement on eligibility was resolved by discussion and a third reviewer for consensus (CM). AI technologies were not used in any part of this review process.

### Data extraction and categorisation

Two reviewers (JOS & CR) extracted data from the full text articles using a custom Excel spreadsheet developed by the research team which included study design, location, method of recruitment, inclusion and exclusion criteria, participant numbers and characteristics, physical assessment and rehabilitation description, PROMs, results and clinical reasoning strategies. The spreadsheet was piloted by the research team to ensure it would extract relevant information prior to full data extraction (Supplemental information 1). A dual extraction method was undertaken to reduce errors in data collection.^34^

The Template for Intervention Description and Replication (TIDieR) checklist^35^ was used by both reviewers (JOS & CR) to extract consistent and detailed information on each of the studies intervention components and mode of delivery. The TIDieR checklist is a validated tool to describe and report interventions in studies^36^ and has been used to extract intervention data from multiple study designs.^37^

As per the PRISMA guidelines for scoping reviews, quality appraisal of included articles for this study was not completed.^30^

### Data synthesis

The extracted data was collated, summarised and reported by the lead author (JOS) and checked by the second reviewer (CR) and discussed with the research team. The TIDieR checklist was used to organise the results of the rehabilitation interventions. As the physical assessments and rehabilitation interventions were comprised of multiple components, these were identified, defined and tabulated by the research team. Clinical Reasoning strategies were assessed on merit subjectively by the lead author and discussed with the second reviewer, based on their own clinical experience of managing patients with NTOS.

## Results

### Study selection

**Figure 1.**
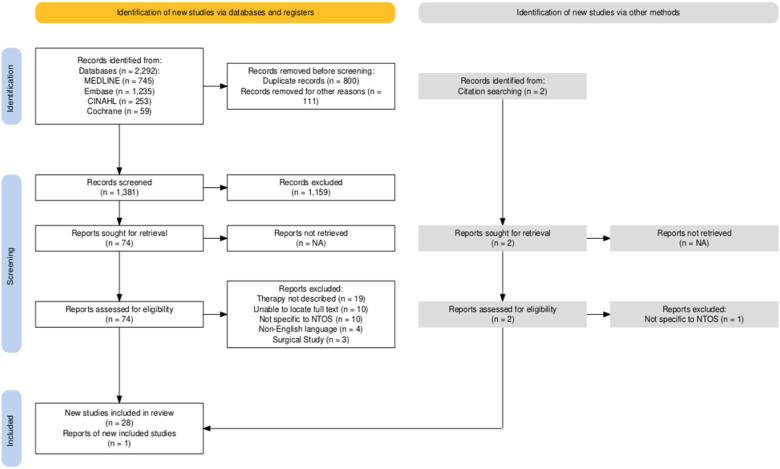
PRISMA flow diagram for study selection.

The results of our search strategy are presented in the PRISMA flow diagram in Figure 1. After omitting duplicates, 1381 records remained for screening. Following title and abstract screening based on the study’s eligibility criteria, 76 studies (*n* = 2 identified from citation tracking) remained for full text retrieval and review. Of these, a further 47 studies were excluded, leaving a final dataset of 29 studies to be included in this review. The main reasons for full text exclusion were as follows; insufficient detail on rehabilitation (*n* = 19), study not specific to NTOS (*n* = 11), non-full text/abstract only (*n* = 10), non-English language (*n* = 4) and surgical studies (*n* = 3).

### Characteristics of included studies

Most of the included studies were literature/narrative reviews or expert opinion pieces (*n* = 13), followed by: prospective studies (*n* = 4), randomised controlled trials (RCT), retrospective analysis and consensus studies (all *n* = 3), case reports (*n* = 2) and one cross sectional study.

Sufficient detail to extract and synthesise information pertaining to the Physical Assessment, Rehabilitation and Clinical Reasoning information was available from 18/29 (62%), 19/29 (66%) and 20/29 (69%) studies respectively. Only seven studies, all review/expert opinion-based articles, had sufficient information on each main theme within the article.^9,19,38–42^ A summary of characteristics of included studies is included in Table 3.

**Table 3.** Characteristics of included studies.

Study reference & country of origin	Study design	Study aims	Sample size	Population characteristics	Intervention/proposed intervention & rationale	Key findings	Elements of study that were used to inform this review (assessment/rehabilitation/clinical reasoning)
Balderman et al. (2017) ^10^ USA	Retrospective analysis of prospective observational cohort single centre data	‘To characterize a prospective observational cohort of patients with NTOS with regard to the prevalence of 14 CDC and results for a broad series of PROMs’	150	NTOS patients confirmed via CORE-TOS CDC	N/A	This study illustrates the relative strengths of 14 CDC and seven PROMs to evaluate patients with NTOS, helping validate the selected CDC.	Clinical reasoning
				107 (71%) female			
				Mean age 37.1 ± 1.1 years (12–66)			
				Mean duration of symptoms-86 (57%) more than 2 years			
						Mean number of positive CDC was 9.6- number of CDC positive were correlated with greater degree of TOP and worse PROMs	
Balderman et al. (2019) ^7^ USA	Prospective observational cohort study	To assess the results of physical therapy management and surgical treatment in a prospective observational cohort of patients with NTOS using PROMs.	150	NTOS patients confirmed via CORE-TOS CDC	‘Physical therapy specific to NTOS’	During a median follow-up >12 months of 130 patients with neurogenic thoracic outlet syndrome, 40 (31%) obtained symptom improvement with physical therapy alone	Treatment/Clinical reasoning
			(40 therapy only group/90 surgical group)	Therapy group- 29 (72%) female	Therapy program consisted of Scalene and Pectoralis muscle stretching and relaxing exercises, with a focus on shoulder girdle and scapular mobility, mechanics, postural improvement, and diaphragmatic breathing, using caution with strengthening, weight training, and the use of resistance bands		
				Mean age 27.7 ± 2.3 (therapy group)			
				Mean duration of symptoms- 21 (52%) more than 2 years			
Berardo et al. (2024) ^48^ USA	Case report	This case report will focus on the use of a patient-centred evaluation approach, and examining personal and environmental factors causing injury, during the initial diagnosis and treatment of non-sport related NTOS in a collegiate ice hockey player	1	Clinical diagnosis of NTOS	‘Physical rehabilitation’	Primary treatment focused on decreasing stress on the anterior chest wall while secondary treatment focused on strengthening the posterior thorax to improve posture.	Assessment/Treatment
				Male	To improve sleep and sleeping position.	Removal from sport never occurred, but symptom alleviation occurred after 3 weeks.	
				Age- 22 years	Alleviate stress on anterior chest wall during shoulder movements and on the neck muscles.	Assessment and treatment specifics described	
				Duration of symptoms- ‘Less than 1 week’	To improve posture via strengthening posterior thorax muscles.		
					Upper limb neural glides to improve NM control to the hands.		
Camporese et al. (2022) ^45^ Italy	Retrospective analysis 2004–2019	Summarises the current evidence about the epidemiology and the pathophysiology of TOS, together with a report of our personal experience in this setting	285 (77 NTOS/136 miscellaneous TOS/38 ‘Vascular TOS (venous and arterial)/22 venous TOS/12 arterial TOS)	238 (84%) female	‘Rehabilitation protocol’	192 (67.4%) improved with rehab	Treatment
				Mean age- 37.9 ± 11.9	‘Restore width of anatomical spaces/address muscle imbalance/postural abnormalities/neural mobilities	Structured rehab SS better outcomes than no therapy.	
					‘Open the thoracic strait’	Higher incidence of TOS in high-risk jobs/worse outcome with concurrent shoulder pathology	
						Treatment program described	
Chim et al. (2024) ^24^ USA	Delphi consensus	Summarises findings from an expert panel of orthopaedic, plastic, and specialised hand surgeons, aiming to reach a consensus among hand surgeons regarding diagnosis and management of NTOS	21 Hand surgeons (INTOS group)	N/A	N/A	17 consensus statements with (>75% agreement) achieved	Treatment/Clinical reasoning
Collins & orpin (2021) ^44^ USA	Review	To describe a thorough history and clinical examination components in NTOS	N/A	N/A	Limiting tensile/compressive loads across the thoracic outlet	The complexity and variable nature of NTOS along with a lack of RCT guidance on management strategies necessitates an individualized approach	Assessment/Treatment
					Maintain patency during functional arm use	A detailed examination of the musculoskeletal components contributing to tension and load across the thoracic outlet is key in creation of an individualized plan of care.	
					Increase strength/endurance/postural awareness		
					Better self-management		
Daley et al. (2021) ^53^ France	Observational prospective study	To compare the isokinetic strength of the shoulder rotators between NTOS patients and healthy controls.	200 (100 NTOS/100 controls)	NTOS diagnosis via CORE-TOS CDC	N/A	NTOS group had SS weaker and less endurance than controls in medial and lateral shoulder rotators	Assessment/Clinical reasoning
				NTOS group-		Weak correlation between disability and weakness e.g.- worse QuickDASH = weaker	
				71 (71%) female			
				Mean age- 39.4 ± 9.6 (22-61)			
				Mean duration of symptoms- 38 ± 34.2 months			
Dengler et al. (2022) ^46^ Germany	Systematic review & consensus	To systematically review the body of evidence and reach a consensus among neurosurgeons experienced in TOS regarding anatomy, diagnosis, and classification	14 surgeons (members of EANS)	Surgeon post graduate experience (mean)- 19.2 years ± 9.8, (range 7-36 years) mean 200 cases±148.9, (range 30-700 cases per surgeon)	N/A	18 consensus statements reached on anatomy, classification and diagnosis	Assessment/Clinical reasoning
						EANS suggest a different classification for NTOS (1/2/3a/3b/3c)	
Fouasson-chailloux et al. (2022) ^54^ France	Observational prospective study	To assess the correlation between the isokinetic shoulder strength and the hand grip and key pinch strength in NTOS patients	130 NTOS patients	NTOS diagnosis via CORE-TOS CDC	N/A	Mean QuickDASH of 59.2±13.9	Clinical reasoning
				94 (72%) female		Hand strength decreased 12.2% & 10% on key pinch compared to asymptomatic side (SS)	
				Mean age- 39.8 ± 9.5 years		Shoulder rotation strength SS weaker on symptomatic side	
				Mean duration of symptoms 3.0 ± 2.7 years			
Garrison et al. (2021) ^49^ USA	Cross sectional study	To compare passive shoulder range of motion (ROM) and anatomic humeral retro torsion (HRT) of baseball players diagnosed with NTOS with a group of healthy, matched controls	106 (53 NTOS/53 controls)	NTOS diagnosis via clinical algorithm of TOS special tests	N/A	Players in the NTOS group had SS less throwing arm ER compared with controls (103.4±10.4 vs 109.6±7.5, respectively; P ¼ .001) and GERD (3.0±9.2 vs 8.8±9.2, respectively; P ¼ .002). TRMdiff was SS greater in NTOS (−11.1±11.1) than in controls (−3.7±9.4) (P < .001).	Assessment/Clinical reasoning
				106 (100%) male			
				Mean age- 17.2 ± 2.4 years			
				Mean duration of symptoms- 7.1 ± 10.9 months			
Glowa & trybulec (2024) ^50^ Poland	Case report	To present the case of a 22-year-old female athlete with the presence of bilateral cervical ribs with simultaneous occurrence of the TOS symptoms, and to present the therapy model and its results	1	NTOS diagnosis via positive functional test and absence of other diagnosis	‘Physiotherapeutic protocol’	Conservative treatment based on the therapy of trigger points, deep tissue massage and rotational manipulations of the cervical spine appear to be an effective form of management of TOS induced by additional cervical ribs	Assessment/Treatment
				Female	Eliminate paraesthesia and to eliminate or minimise pain in back and shoulder girdle muscles		
				Age- 22			
				Duration of symptoms- not stated			
Goeteyn et al. (2022) ^8^ Netherlands	RCT	To objectify the effect of thoracic outlet decompression (TOD)	50 NTOS patients (25 surgical/25 continued therapy)	NTOS diagnosis via SVS CDC	‘TOS dedicated Physio’	After 3 months of continued therapy all subjects in continued therapy group had SS worse PROMs compared to surgical group, and all chose to switch to surgical arm.	Treatment/Clinical reasoning
		Patients with a diagnosis of NTOS refractory to conservative therapy were randomised to one of two intervention arms, receiving either a transaxillary thoracic outlet decompression (TA-TOD) or continued conservative treatment. After 3 months, the conservative treated group was also offered a TA-TOD		Surgical group 23 (96%) female therapy group 16 (73%) female	The goal of the TOS dedicated physiotherapy is improvement of the patient’s posture by strengthening exercises and lengthening of the shoulder girdle muscles	6 months after randomisation no SS difference between groups was observed.	
				Mean age- surgical group 37.7 ± 9.8 years therapy group 40.0 ± 9.5 years		TA-TOD for NTOS is effective in patients who do not respond to conservative treatment.	
				Mean duration of symptoms- surgical group 1.5 years (1-2.9) therapy group 3.0 years (1.8-4.4)			
Hisamoto (2021) ^38^ USA	Expert opinion	N/A	N/A	NTOS diagnosed as per SVS CDC	‘HI-TOP: The proactive approach- hisamoto- illig thoracic outlet programme’	Comprehensive assessment described	Assessment/Treatment/Clinical reasoning
					Active rehabilitation treatment goals include postural alignment, scapular stabilization, and proper shoulder positioning, all of which play an important role in alleviation of symptoms and, ideally, return to functional activities. Adjunct therapies are utilized to ensure reduction in symptoms for progression of the active exercise program	Staged treatment approach described	
						Chronic tightness of scalene/PM/SA, causes adapted lengthening of MT & LT- can lead to ‘upper crossed syndrome’ - leading to SCM/suboccipital, UT/LS/pecs tightness & lengthened/weak- deep neck flexors/MT & LT, SA, rhomboids/posterior chain of shoulder	
						States 75% of NTOS patients will also have compression at subcoracoid space	
Hock et al. (2024) ^22^ USA	Review/Expert opinion	N/A	N/A	N/A	‘Rehabilitation protocol’	Comprehensive assessment described	Assessment/Treatment
					Improve scapula control, improve joint mobility. Improving diaphragmatic breathing can help decrease hypertrophy of accessory breathing muscles primary. Rehab goal = limit tensile/compressive loads across the thoracic outlet region	Staged treatment approach described	
						Suggested 4-6 months trial of conservative management	
						Uses a strength of recommendation taxonomy	
Kuwayama et al. (2017) ^43^ USA	Review	We describe some approaches to aid in identifying patients who would be expected to benefit from surgical intervention for NTOS.	N/A	N/A	Treatment plan can be constructed to influence the narrow passageways through which the neurovascular tissue has become irritated. Treatment involves a comprehensive approach of manual therapy directed at bone or joint position, soft tissue mobilization, and therapeutic exercise focusing on recruitment of stabilisation-based musculature and inhibition of over-utilized muscles	Comprehensive assessment described	Assessment/Treatment
		We describe the role of physical examination, physical therapy, and imaging in the evaluation and diagnosis of NTOS.				Suggests 6 weeks of conservative treatment input- and can then expect results	
Levine et al. (2018) ^39^ USA	Review	A comprehensive study of shoulder anatomy and biomechanics, and knowledge of the benefits of exercise, may help clinicians and healthcare practitioners determine the most appropriate treatment plan for an individual with TOS	N/A	N/A	‘Exercise’ initially- incorporate shoulder movements ranging from 0 to 30° flexion in scaption plane- progress to 45-90° flexion and functional overhead tasks. Start targeting - Scapular muscles (MT/LT and Rhomboids) as progress made strengthen SA- stretching of Scalenes/Pec muscles whilst strengthening neck (Neck erectors, Rhomboids, LT) should be an area of focus	Describes shoulder functional anatomy and mechanics and relates to TOS symptoms.	Assessment/Treatment/Clinical reasoning
						Describes exercises for NTOS	
						Describes length-tension relationship between FHP/Protracted scapula and the effect of this on closing the thoracic outlet	
Li et al. (2021) ^9^ USA	Review	To review the epidemiology, aetiology, relevant anatomy, clinical presentations, diagnosis, and management of thoracic outlet syndrome	N/A	N/A	‘Conservative management’	Comprehensive assessment described	Assessment/Treatment/Clinical reasoning
					Aims to alleviate neurovascular strain to reduce symptom severity and frequency through non-invasive means	Described exercise and therapy adjuncts for NTOS (taping, braces, massage etc)	
						Describes positive and negative prognostic factors for physiotherapy	
Luu et al. (2022) ^21^ Canada	Review	Analyse the literature, from inception to March 2021, on rehabilitative exercises for true and disputed NTOS, to provide a broad and comprehensive overview of different exercise protocols our secondary aim was to review the clinical reasoning behind different exercise protocols which will help guide rehabilitation clinicians in the management of NTOS	47 articles included from 1975 to 2021	True or disputed NTOS	N/A	*Peets rational/Britts-* restore muscle balance and achieve postural correction by strengthening muscles that open the thoracic outlet by raising the shoulder girdle and stretching muscles that close the thoracic outlet	Clinical reasoning
						*Watson et al-* ex to address dropped shoulder and elevate the shoulder girdle to decompress the TO and restore scapula control nerve gliding (crosby)- proposed that these exercises would help to decrease intraneural pressure, minimize scarring, decrease extrinsic pressure and reduce intrinsic irritation of the surrounding neurovascular structures-	
Olson et al. (2023) ^55^ USA	Retrospective chart review	We sought to identify typical presenting symptoms and common findings on diagnostic workup, in addition to evaluating rates of return to play following various treatment interventions	36 NTOS division 1 athletes	Clinical diagnosis of NTOS	‘TOS focused physical therapy’ Edgelow neurovascular entrapment self treatment protocol	Comprehensive assessment described, including potential risk factors	Assessment/Clinical reasoning
			From 2000 to 2020	23 (64%) female	Anterior Scalene botox injection if inadequate or temporary relief with PT	Minimal detail regarding therapy treatment	
				Mean age- 19.9 ± 1.2 years	Surgical decompression if inadequate or temporary relief with botox	42% of athletes could continue in sport despite symptoms- of those who had to cease playing- 12% returned with PT alone & 42% able to return post botox	
				Mean duration of symptoms- <3 months 64% >1 year 19%	**If no response to botox or PT then patients were not offered surgery as botox was used as a diagnostic & prognostic tool**		
				Dominant arm- 81%			
Ortac et al. (2020) ^51^ Turkey	RCT	To assess the effects of kinesio taping (KT) on pain, paraesthesia, functional status, and overall health status in patients with symptomatic thoracic outlet syndrome (sTOS)	Termed ‘sTOS’ in this study- but consistent with NTOS in other studies 60 participants (30- sTOS/30 control)	Kinesio tape group- 26 (87%) female control group- 25 (83%) female	Kinesio taping (3 times for 4 days- 12 days total) vs placebo taping	Diagnosis not strictly SVS or CORE-TOS CDC	Treatment
				Mean age- kinesio tape group 33.5 (22-46) control group 26.0 (20-43)	To relieve tension over the compression site, the muscle inhibition technique was applied. The application targeted four muscles: Subclavian, Pectoralis Minor, Biceps, and Anterior Scalene.	SS differences in most PROMs favouring KT group over control group.	
				Mean duration of symptoms- not stated ‘at least 3 months’		However, on removal of tape- measures returned to baseline	
						Described taping procedure sufficient and provided video	
Panther et al. (2022) ^40^ USA	Review	In this review, we outline the diagnostic tests and treatment options for TOS to better guide clinicians in recognizing and treating vascular TOS and objectively verifiable forms of neurogenic TOS	N/A	N/A	‘Disease specific PT’ ‘reduce pressure on the involved neurovascular structures’	Described comprehensive assessment	Assessment/Treatment/Clinical reasoning
						Describes some prognostic factors for response to therapy	
						Minimal conservative treatment points	
						Good table for clinical tests	
Pesser et al. (2021) ^11^ Netherlands	Prospective observational cohort study	The results of a standardised multidisciplinary care pathway for NTOS based on the north american SVS reporting standards for NTOS are reported	476 NTOS patients	NTOS diagnosis via SVS CDC	All pts with NTOS underwent physio as primary rx- programme consisted of posture evaluation and improvement, shoulder girdle therapy and focused scapula mobility therapy	Provides a good framework to design a trial regarding SVS reporting guidelines	Treatment/Clinical reasoning
				Sex and mean age not stated for therapy group only surgical group		39.1% improved with therapy- but didn’t report the characteristics of who these were- and were not included in follow up data	
						Used scalene blocks in all patients suspected of NTOS	
Rochkind et al. (2023) ^47^ Israel	Systematic review & consensus	To document consensus and controversy on NTOS management, with emphasis on timing and types of surgical and nonsurgical NTOS treatment, and to support patient counselling and clinical decision-making within the neurosurgical community	15 surgeons (members of EANS)	Surgeon post graduate experience (mean)- 19.1 years ± 10.6, (range 7-36 years) mean 189 cases±204, (range 30-700 cases per surgeon)	N/A	Fourteen statements on nonsurgical NTOS treatment, timing, and type of surgical therapy were developed. Within our expert group, the agreement rate was high with a mean of 97.8% (±0.04) for each statement, ranging between 86.7% and 100%	Clinical reasoning
						Some potentially useful clinical regarding points regarding prognosis	
Taskaynatan et al. (2007) ^52^ Turkey	RCT	The aim of this study was to investigate the effects of cervical traction and exercise in addition to moist heat in TOS	40 TOS patients	Clinical diagnosis of TOS intervention group- 16 (80%) female control group- 8 (40%) female	Intervention-Cervical traction, hot pack and exercise	Treatment approach described	Treatment
				Mean age- 27.2 ± 7.5 years	Control- Hot pack and exercise program	Described as ‘TOS’ within this study but presentation consistent with NTOS	
				Mean duration of symptoms- 10.7 ± 10 months	Decreasing the compression on the brachial plexus, restoring neural mobility, and correcting muscle imbalance in the cervicoscapular region. Cervical traction to stretch the neck muscles. Increase awareness of muscular tension and postural imbalance	Used response to provocation tests between groups as a standard of improvement- but didn’t discuss the test responses in detail	
Troyer et al. (2023) ^25^ USA	Review	To review and summarize the literature on the clinical presentation, exam signs, diagnostic tools, and available treatments for NTOS with particular emphasis on overhead and throwing athletes	N/A	N/A	Physical rehabilitation is the generally accepted initial treatment plan. Rehabilitation for NTOS should focus on Scalene and Pectoralis Minor relaxation and stretching and emphasise shoulder girdle, scapular, and glenohumeral mechanics (level IV)	Comprehensive assessment described	Assessment
						Insufficient detail on treatment	
Vanti et al. (2007) ^20^ Italy	Review	To evaluate the efficacy of conservative treatment in TOS with reference to physiotherapy, orthotics, and taping and to make general recommendations for conservative treatment.	N/A	N/A	N/A	Good summary of history of conservative management is provided	Assessment/Clinical reasoning
						Assessment is described	
						Makes 10 recommendations for conservative care- providing some clinical reasoning points	
Wagner et al. (2023) ^41^ USA	Expert opinion/diagnostic algorithm	To present our standardized diagnostic algorithm for NTOS and surgical technique for arthroscopic suprascapular neurolysis, pectoralis minor release, brachial plexus neurolysis, and infraclavicular thoracic outlet decompression	N/A	N/A	‘Supervised physical therapy’ correction of Scapulothoracic motion (chronic protraction), rotator cuff and periscapular muscle strengthening and postural retraining	Comprehensive assessment described	Assessment/Treatment/Clinical reasoning
						Treatment approach (conservative and post-operative described)	
						Clinical reasoning points suggested (to look out for SSN and PMS pathology.	
Warrick & Davis (2021) ^42^ USA	Review	To review the key points of NTOS evaluation and management, as well as update readers on management of athletes with NTOS	N/A	N/A	‘Physical therapy’ physical therapy focuses on improving posture, biomechanics, modifying activities, stretching tightened muscles, stabilizing the scapula, strengthening weakened muscles, educating athletes about the goals of care, and developing a home exercise program. ‘Stretching muscles that close the TO and strengthen those that open it'	Assessment described	Assessment/Treatment/Clinical reasoning
						Treatment approach described	
						Clinical reasoning points discussed	
Watson et al. (2010) ^19^ Australia	Expert opinion	The purpose of this paper is to comprehensively describe one treatment approach for the conservative management of TOS.	N/A	N/A	‘Rehabilitation program’ the management approach described in this article is designed to elevate the shoulder girdle and restore scapula control	Comprehensive assessment described	Assessment/Treatment/Clinical reasoning
						Comprehensive staged treatment approach described for scapula rehabilitation along with therapy adjuncts	
						Clinical reasoning points suggested	

CDC: clinical diagnostic criteria; CORE-TOS: consortium of research and education on thoracic outlet syndrome; EANS: the European association of neurosurgical societies; ER: external rotation; FHP: forward head posture; GERD: glenohumeral external rotation deficit; INTOS: international neurogenic thoracic outlet syndrome hand surgery workgroup; LS: levator scapulae; LT: Lower fibres of trapezius; MDT: multidisciplinary team; MT: middle fibres of trapezius; N/A: not applicable; NM: neuromuscular; NTOS: neurogenic thoracic outlet syndrome; PM: pectoralis minor; PMS: pectoralis minor syndrome; PROMs: patient reported outcome measures;  PT: physical/physiotherapy; RCT: randomised control trial; Rx: treatment; SA: serratus anterior; SCM: sternocleidomastoid; SS: statistically significant; SSN: suprascapular nerve; SVS: society of vascular surgeons; TENS: transcutaneous electric nerve stimulation; TO: thoracic outlet; TOP: tender on palpation; TRMdiff: total rotational movement difference; USS: ultrasound.

## Review findings

### Primary aim-physical assessment and rehabilitation components and clinical reasoning strategies

#### Physical assessment

The most prevalent physical assessment components encountered were provocation tests- *Roos/EAST* (16/18 89%), *ULTT* (14/18 77%) and *Adson’s* (10/18 56%). Additionally, 11 (61%) studies mentioned, *Palpation of pertinent structures (pectoralis muscles/scalene/supraclavicular space/subcoracoid space),* whilst 10 studies (56%) mentioned*; Posture assessment* and *Scapulothoracic assessment*.

Less frequently encountered physical assessment components such as, *first rib mobility* (*n* = 3)^9,20,43^ and *breathing assessment (n = 1)* were also noted.^44^ Three studies,^8,10,38^ suggest that their physical assessment of NTOS is based on patients fulfilling existing CDC’s.^17^

**Figure 2.**
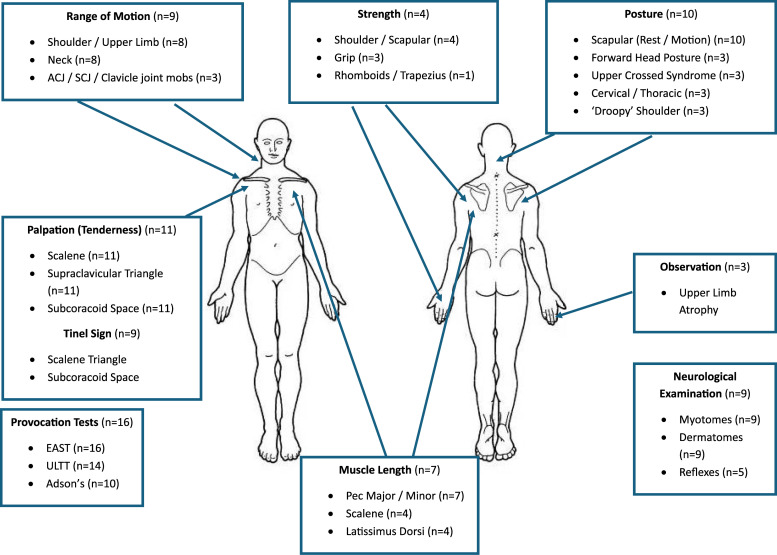
A body chart to visually represent a comprehensive physical assessment of NTOS, based on the content of included studies (*n* = number of studies each component included). Further information in supplementary information 2. ACJ: acromial-clavicular joint; SCJ: sternoclavicular joint; mobs: mobilisations; EAST: elevated arm stress test; ULL: upper limb tension test; Pec: pectoralis.

Due to the overlap in the description of physical assessment components, further coding and synthesis was conducted to allow a visual representation of a comprehensive physical assessment of NTOS, based on the findings of the included studies (Figure 2). Further information on assessment is available in Supplemental Information 2.

### Rehabilitation

The description of rehabilitation interventions based on a modified version of the TIDieR checklist are summarised and presented in Table 4. Further information is available in Supplemental Information 2.

**Table 4.** Summary of rehabilitation interventions of included studies, using a modified version of the TIDieR checklist.

TIDieR checklist item	Description (*n* = number of studies)
**Why? (Rationale/Goal)**	• Improve ROM, neural mobility, muscle imbalance, strength, endurance (*n* = 12)^11,22,25,38,39,41,42,44,45,48,49,52^
	• Reduce compression/stress/sensitivity of neurovascular structures (*n* = 10)^9,22,40,42-44,48,49,51,52^
	• Improve postural abnormalities/awareness (*n* = 10)^8,11,38,39,41,42,44,45,48,49^
	• Restore width of anatomical spaces/‘open’ the thoracic outlet (*n* = 6)^8,22,42-45^
	• Improve/restore scapular control (*n* = 6)^11,19,22,25,41,42^
**What? (Materials)**	• Advice/education- activity modification, ergonomics, posture advice (*n* = 9)^9,19,38,42,44,45,48,51,52^
	• Postural brace/taping (*n* = 8)^9,19,22,38,41,44,48,51^
	• Home exercise programme (*n* = 5)^11,19,38,39,52^
	• Exercise related e.g. resistance bands, dumbbells, foam roller, gym ball, CV equipment (*n* = 5)^19,38,44,45,48^
	• Mirrors, video, biofeedback (*n* = 1)^19^
	• Heat/cold/Instrument assisted soft tissue mobilisation (IASTM)/electrotherapy (*n* = 1)^38^
**What? (Procedures)**	• **Exercise (*****n*** **= 17)**
	• Stretching/Mobility/Elongation (*n* = 15)^7–9,11,19,22,24,38,39,41,42,44,48,50,52^
	• Strengthening (*n* = 14)^48,45,24,44,8,38,22,39,9,40,52,41,42,19^
	• Neural mobility (*n* = 7)^22,24,38,40,42,44,48^
	• Diaphragmatic breathing (*n* = 6)^7,22,38,44,45,52^
	• Cardiovascular (*n* = 1)^44^
	• **Posture improvement (*****n*** **= 13)**
	• Via exercise (*n* = 11)^7,11,24,38,40,42-45,48,52^
	• Brace (*n* = 1)^48^
	• Via education (*n* = 1)^51^
	• No further details (*n* = 1)^41^
	• **Manual therapy (*****n*** **= 10)**
	• Soft tissue massage (*n* = 7)^9,19,38,43,44,48,50^
	• Joint mobilisations (*n* = 5)^19,22,38,44,45^
	• Therapist led passive stretches (*n* = 3)^38,44,45^
	• Trigger point therapy (*n* = 2)^24,50^
	• Manipulations,^50^ cupping,^38^ dry needling,^22^ traction^52^ (all *n* = 1)
	• **Adjuncts (*****n*** **= 8)**
	• Taping (*n* = 6)^9,19,22,38,44,38^
	• Posture brace (*n* = 4)^9,19,41,48^
	• **Activity modification (*****n*** **= 7)**
	• Inhibit anterior chest/neck muscles or pushing exercises (*n* = 2)^44,45^
	• Stop overhead activity,^45^ avoid shoulder ‘drooping’,^24^ sleep advice,^44^ caution with weights/strengthening,^7^ avoidance of irritating positions^52^ (all *n* = 1)
	• Avoid first rib manipulations (*n* = 1)^8^
	• No further details (*n* = 1)^42^
	• **Psychosocial informed treatment (*****n*** **= 1)**
	• CBT/motivational interviewing/self-efficacy/adherence/understanding/meditation/relaxation (*n* = 1)^44^
**When & How much? (Dose, frequency, duration)**	**When**
	• Occurred when most participants had symptoms present for more than 2 years (*n* = 4)^7,8,10,11^
	• 2 studies both RCTs, had participants with symptoms for at least 3 months^51,52^
	• 2 studies had symptoms less than 3 months^48,55^ with a case study involving a patient with symptoms lasting less than a week^55^
	**Intervention length**
	• 6 months was the most reported timeframe suggested for therapy (n = 6)^9,24,39,40,42^ all were review/consensus articles except one retrospective analysis by Camporese et al.^45^
	• 3 months of continued therapy for patients previously refractory to improvement with therapy was delivered in an RCT^8^
	• 6–12 Weeks was proposed by two expert opinion articles^19,38^ and delivered in one prospective observational study of 476 NTOS patients^11^
	• Balderman et al.^7^- provided 6 weeks of therapy to 150 NTOS patients in a prospective observational study; 6 weeks was also proposed by a review article^43^
	• Two case reports both reported treatments lasting 3 weeks,^48,50^ and an RCT involving cervical traction lasting for 2 weeks^52^
	• An RCT involving kinesio taping lasted for 12 days^51^
	**Frequency/session duration**
	• Nil relevant information on dosage in 10 articles^7,8,9,24,39,41-45^
	• An RCT^52^ delivered 10 sessions, 5 days a week for 35-45 min of hot packs and cervical traction- 20 s on- 10 s off with 10–15 kg of traction weight
	• Another RCT^51^ provided 3 separate sessions of kinesio taping for 4 days each
	• A prospective observational study, recommended daily unsupervised exercise, and 1 session per week of supervised input^11^
	• 2 studies provided dosage information for stretching exercises- one stating 4 sets of 30 s holds,^48^ the other 20 s holds, for 3–5 repetitions^38^
	• For strengthening exercises- 2–3 sets of 8–15 repetitions were recommended.^48^ 4 articles advised starting with a ‘high reps, low weight’ approach when performing strengthening exercises^45382242^
	• For isometric/postural setting exercises, hisamoto^38^ recommended 5 s holds, for 5 repetitions for 2 sets each day
	• Nerve gliding exercises were recommended with caution to begin with, progressing up to 25 repetitions up to 2–5 times daily^38^
	• Watson^19^ recommended a dosage of 20 reps x3 sets per day for all type of exercises related to NTOS
	• Cardiovascular exercise of 30 min per day was recommended by 1 study^44^
	• 2 expert opinion articles, recommended a staged approach to exercise progression^19,38^

**Figure 3.**
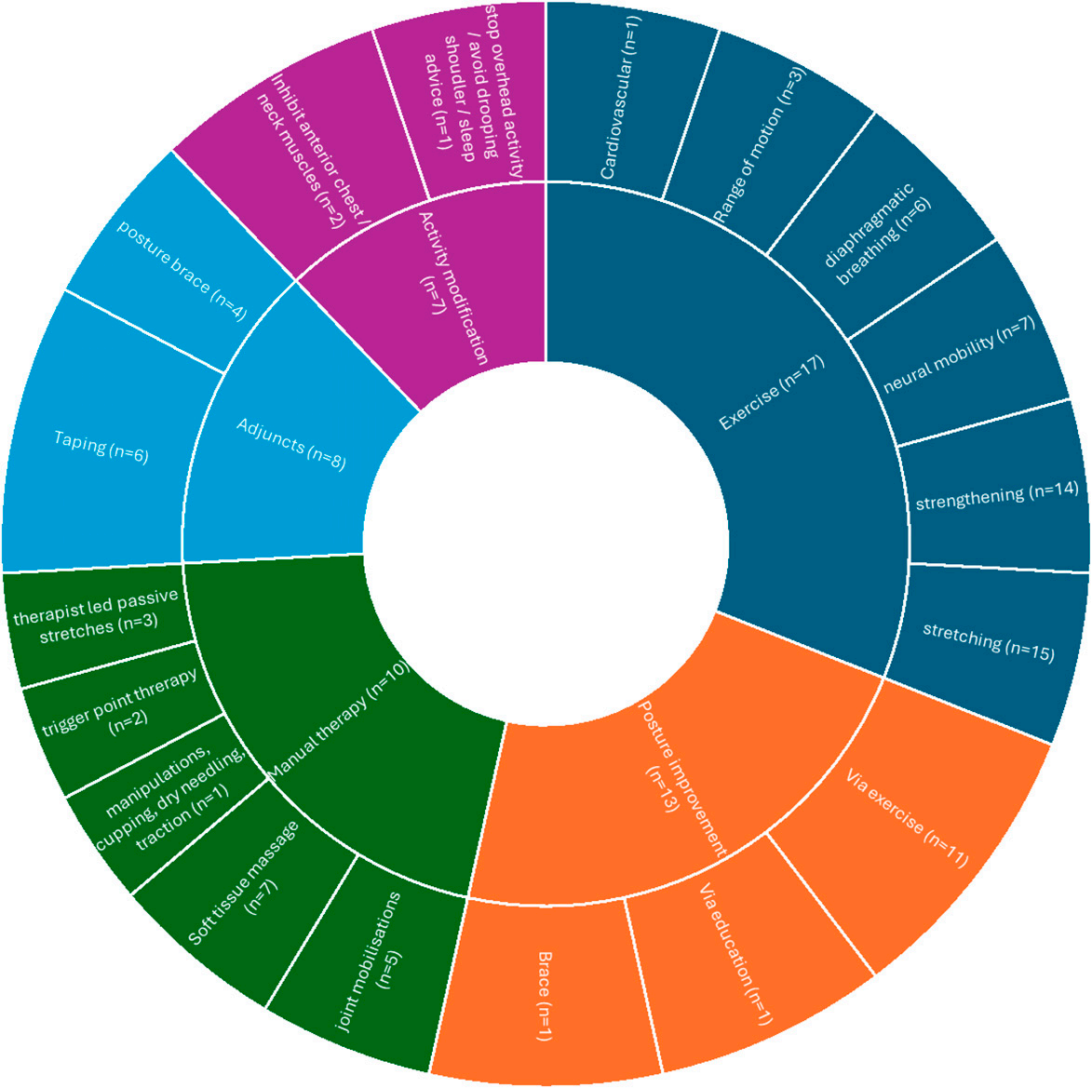
A nested pie chart to represent the main rehabilitation elements and their components found in the included studies, and their frequency (*n* = ). The size of the inner segments represented the frequency of the element being encountered within the studies.

Most of the included studies describing rehabilitation interventions (17/19) included exercise as a core element. Stretching (*n* = 15), strengthening (*n* = 14), neural mobility (*n* = 7) and diaphragmatic breathing (*n* = 6) were the most prevalent exercise components encountered. Additional rehabilitation interventions were: ‘Posture Improvement’ (*n* = 13), ‘Manual Therapy’ (*n* = 10), ‘Adjuncts’ (*n* = 8) such as taping or braces, and advice on ‘Activity Modification’ (*n* = 7). Only one study^44^ proposed using a ‘Psychosocial informed treatment’. Figure 3 provides more details.

Most interventions were provided by a physical or physiotherapist (*n* = 14), via face-to-face means (*n* = 11) in a clinic setting (*n* = 11). Patients receiving treatment often had chronic symptoms of at least 2 years (*n* = 4). Information on intervention frequency, duration and dosage was typically sparsely described within the included studies, with nil relevant information on dosage in 10 articles. When describing strengthening exercises for NTOS, a ‘high repetition, low weight’ approach was suggested in four studies.^22,38,42,45^

The Scalene and Pectoral muscles (*n* = 10) were most frequently mentioned muscles when describing stretching exercises. Scapular Stabilisation (*n* = 9) was the most common feature of strengthening exercises, followed by mid-lower Trapezius and Serratus Anterior (*n* = 5). Minimal detail for neural mobility exercises other than ‘upper limb neural glides’ (*n* = 6) was provided, and no further details concerning diaphragmatic breathing exercises were provided by any of the six studies. Further information on specific exercises that were mentioned in the included studies can be found in Supplemental Information 2.

### Clinical reasoning

#### Prognosis

Balderman et al., in their two studies^7,10^ of 150 NTOS patients, displayed some significant differences between the groups of patients that improved with rehabilitation alone (31%), and those that did not (69%). The group that improved with rehabilitation were on average less tender to palpation (1.7 ± 0.1 vs 2.0 ± 0.1 *p* < 0.05), had less positive CDC signs (9 ± 0.3 vs 10.1 ± 0.1 *p* < 0.05), less severe Cervical Brachial Symptom Questionnaire (CBSQ) (68.0 ± 4.1 vs 78.0 ± 2.7 (*p* < 0.05) and Short Form 12 (SF-12) physical component (35.6 ± 1.5 vs 32.0 ± 0.8 *p* < 0.05) scores and could tolerate a longer EAST test before failure (103 s ± 10.2 vs 97 s ± 6.3 *p* > 0.05). However, there was no between group differences noted in age, gender, symptom duration, previous injury, QuickDASH or other PROMS.

Two review articles^9,20^ proposed that patient compliance to rehabilitation, resulting in lasting lifestyle/postural modifications and a sedentary job were positive prognostic factors for response to rehabilitation in NTOS whilst obesity, depression, previous upper limb trauma and chronicity of symptoms were negative factors.

#### NTOS management decisions

A two-part consensus study by members of the European Association of Neurosurgical Societies (EANS) proposed a subclassification of NTOS to guide clinical management decisions.^46,47^

The group proposed that patients with atrophy/objective weakness (NTOS 1) should be assessed urgently for potential surgery. Conservative management including rehabilitation should be the first treatment for NTOS 2 & 3a patients and failing that, may progress to surgery. Whereas NOTS 3b & 3c patients should only progress from conservative care to surgery in rare circumstances and these patients would need to be counselled on the risks of potentially unsuccessful surgery. A further consensus study, from the International Neurogenic Thoracic Outlet Syndrome (INTOS) Hand Surgery workgroup,^24^ agreed with a 3–6-months conservative care first approach for all NTOS patients, except those with atrophy or weakness, but did not fully support the EANS subclassification. Further information is available in Supplemental Information 2

### Secondary aim-diagnosis and measurement

#### Diagnosis

As diagnosing NTOS can be complex, a secondary aim of this study was to examine how the included studies chose to clarify the diagnosis of NTOS.

Of the 16/29 (55%) of studies in this review to specifically discuss diagnosis of NTOS, 56% (9/16) of them quoted either the Society of Vascular Surgeons (SVS) CDC (*n* = 6)^17^ or the Consortium of Research and Education on Thoracic Outlet Syndrome (CORE-TOS) CDC (*n* = 6).^16^ Two studies^7,24^ referenced both. Other studies (*n* = 5) were less specific, but their description encompassed elements of both previously mentioned CDCs, for example the requirement of positive provocation tests (Elevated Arm Stress Test (EAST), Upper Limb Tension Test (ULTT) and Adson’s)^46,48,49^ and the absence of other more likely diagnoses.^40,50^

Two studies, both RCT’s,^51,52^ offered their own criteria for the diagnosing NTOS. Ortac et al.’s (2020) criteria, while less comprehensive, would still align with the CDC proposed by Thompson.^16^ However, Taskaynatan et al.’s^52^ would not sufficiently justify the diagnosis of NTOS according to more widely accepted CDC’s, potentially questioning the validity of their study findings.

Additionally, Balderman et al.^10^ described the most prevalent elements of the CORE-TOS CDC found within their study (n = 150 patients). Elements encountered in more than 90% of patients included: pain (99%), symptoms *exacerbated by elevation* (97%), tenderness *to palpation of scalene triangle/subcoracoid space* (96%), numbness*, paraesthesia or weakness in arm and/or hand* (94%) and *positive EAST test* (94%). The least prevalent positive elements were a *history of previous clavicle/first rib fracture or presence of a cervical rib* (8%), *previous cervical or peripheral nerve surgery* (20%), *previous treatment for ipsilateral TOS* (21%) and *weak handgrip/hand intrinsic atrophy* (23%).

Two consensus studies^24,46^ both emphasised the importance of patient history and physical examination in the diagnosis of NTOS, along with arm symptoms referring along the C8/T1 distribution, which is also proposed by multiple other studies.^25,38,42,48,52^

#### Measurement

Twelve out of 18 (67%) studies used/proposed to use the QuickDASH. The CBSQ was less frequently observed (8/18 44%), with the TOS Disability score featuring in only 2 of the 18 studies.^8,11^ The Short Form 12 (SF-12) was used 6 times, whereas the Pain Catastrophising Scale and the Zung Self-Rating Depression score were only encountered twice.^7,10^ Four studies assessed functional changes such as isokinetic strength of shoulder rotators,^53^ grip strength,^54^ changes in range of motion or tenderness to palpation^50^ and time to return to play.^55^ Further information regarding measurement is available in Supplemental Information 2.

## Discussion

The primary aim of this scoping review was to identify and describe the components of physical assessments, rehabilitation and clinical reasoning strategies that may assist therapists in managing adults with NTOS. The secondary aim was to examine approaches to diagnosing NTOS and the use of PROMs. This review examined literature published since 2000, with most studies (*n* = 22) published since 2020.

This review highlights the wide range of assessment and rehabilitation components associated with the therapy management of NTOS. A continued emphasis on the biomedical model is evident in the assessment and rehabilitation of NTOS, with many studies stating their aim of treatment is to reduce ‘stress/compression’ of neurovascular structures and ‘open’ the thoracic outlet. This echoes the findings of the 2022 scoping review on exercise and NTOS.^21^

### Assessment

The most frequently encountered physical assessment components appear to focus on confirming a diagnosis of NTOS, rather than identifying targets for therapy intervention. Commonly used methods include provocation tests (EAST *n* = 16) and palpation of pertinent structures (*n* = 11). Postural assessment also features prominently (*n* = 10), particularly in relation to scapular positioning. This emphasis is likely tied to the perceived impact of scapular alignment on thoracic outlet space; however, this association remains largely anecdotal.^19^ Notably, only one article proposes an objective method for measuring scapular position,^19^ and none of the studies provide follow-up data assessing postural changes as an outcome of treatment. A similar pattern is seen in the assessment of muscle length in pectoral (*n* = 7) and scalene muscles (*n* = 4). While these assessments are often based on presumed symptom contributors, they are rarely conducted in an objective or reproducible manner, nor are they re-evaluated following interventions. This raises two important questions: firstly, ‘Do we need to assess these aspects if we are not going to re-assess them?' and secondly ‘How do we know the perceived effect that rehabilitation is having on NTOS patients?'.

Logic modelling has been described as a practical way of representing theoretical data on how a treatment programme may be effective.^56^ Proposing a logic model to illustrate the potential effects of rehabilitation on NTOS may help address current gaps in understanding and further our knowledge of both the underlying causes of NTOS symptoms and the impact of specific interventions. Several studies included in this review provide potential starting points for such a model.

Two prospective observational studies assessed and identified that NTOS patients had weaker grip strength on the symptomatic side^54^ and significantly decreased shoulder rotation strength compared to controls.^53^ Additionally, Garrison, Hannon and Conway^49^ identified that NTOS patients had significantly reduced total shoulder rotational movement compared to controls in their cohort of baseball players. Tenderness to palpation is another physical assessment component that is commonly encountered, although reliably being able to objectify this may be challenging. In general, the physical assessment components reported in this review align with a recent UK survey of NTOS clinicians.^15^

Future research should prioritize consistent, measurable assessment criteria to support logic model development and standardise therapy approaches.

### Rehabilitation

Rehabilitation interventions for NTOS were vast, with considerable overlap in their descriptions. This review has summarised the main elements, their components and their potential therapeutic targets, which is hopefully helpful to the clinician when building a rehabilitation programme. Exercise was the most frequent element encountered (*n* = 17), with both stretching (*n* = 15) and strengthening (*n* = 14) exercises targeting the neck and shoulder girdle. These interventions were commonly proposed to “decompress” the thoracic outlet. Neural mobility exercises (*n* = 7) were also included to reduce sensitivity in the neurovascular structures. Postural improvement was a feature of several studies included in this review (*n* = 13), with most (*n* = 11) suggesting exercise as the primary method to achieve this. However, few studies either assessed or re-tested functional markers before and after treatment, making it difficult to draw firm conclusions about the efficacy of these interventions. Only four studies in this review reported objective functional markers in NTOS patients.^50,53–55^ PROMs, such as the QuickDASH (*n* = 12), CBSQ (*n* = 8) and SF-12 (*n* = 6) dominated the outcome measures, perhaps due to the increasing awareness of and adherence to the SVS reporting guidelines.^17^

### Clinical reasoning

Inconsistencies were identified within the included studies regarding the justification for rehabilitation components for NTOS. Studies cautioned against the use of neural mobility^44^ and resistance^7^ exercises for fear of exacerbating symptoms, despite both featuring prominently in most rehabilitation descriptions. Additionally, manual therapy to the first rib was encouraged in five studies,^19,22,38,44,45^ but excluded from a rehabilitation package in an RCT^8^ as they felt it may aggravate pain. The scapula also emerged as a prominent rehabilitation focus within the included articles in this study (*n* = 9), mirroring its frequent role in posture assessment. Studies proposed that improving the stabilisation of the scapula can help decrease symptoms, but how this was purported to be achieved differed. Several studies proposed completing scapular depression/retraction exercises,^24,38,45,48^ whereas two studies advised against them.^19,44^ The latter argued that excessive scapular depression may increase compression or mechanical stress on neurovascular structures within the thoracic outlet, instead promoting exercises that enhance scapular elevation and upward rotation.

In the authors' opinion, both approaches may be valid. For example, patients with shoulder protraction and forward head posture may benefit from retraction exercises, while strengthening into elevation and upward rotation supports accepted functional scapular kinematics required for efficient shoulder function.^57^

Additionally, there are notable discrepancies between the rehabilitation programmes delivered in primary studies and those proposed in review articles. A six month duration of rehabilitation was the most recommended timeframe across five review articles.^9,24,39,40,42^ However, none of the larger prospective studies provided rehabilitation over this timeframe (Table 4).^7,8,11^

Shorter durations may reflect research constraints, though 6 months is commonplace in NHS practice in the authors' experience, and this is supported by UK survey data.^15^

Only one study^44^ proposed psychologically informed treatment for NTOS, despite its widespread use in the management of chronic pain conditions.^58^ This highlights a persistent biomedical bias in NTOS care. Incorporating holistic PROMs could guide more comprehensive therapies, for which excellent long-term results have been recently published for chronic disabling low back pain.^59^

Predicting which NTOS patients are likely to benefit from therapy would be highly advantageous, both for optimising patient outcomes and for the efficient allocation of healthcare resources. Balderman et al.^7^ identified characteristics associated with a favourable response to rehabilitation compared to those who eventually opted for surgery. In general, patients who responded positively had less severe symptoms, including reduced tenderness to palpation, less severe CBSQ and SF-12 (Physical Component) scores, fewer positive clinical diagnostic criteria, and a trend to a greater tolerance of the EAST test. This observation is consistent with our clinical experience and is supported by findings in other upper limb conditions, where less severe symptoms have been identified as positive prognostic indicators for rehabilitation response.^60^ Further cohort studies focusing on NTOS patients undergoing rehabilitation would help advance this area of research. Notably, some studies included in this review did not provide detailed profiles of patients who responded positively to rehabilitation,^11^ limiting our ability to draw stronger conclusions about predictive factors.

Finally, the classification and terminology surrounding NTOS remains inconsistent and, at times, confusing. Multiple variations, such as “true,” “disputed,” “symptomatic,” and “miscellaneous” were still evident in the studies reviewed, despite the presence of generally accepted reporting standards.^17^ The EANS consensus study^47^ proposed a new classification system (NTOS 1, 2, 3a/b/c), which may offer some value, particularly in identifying patients who may not be suitable candidates for rehabilitation, such as those with NTOS 1, who present with objective intrinsic muscle atrophy and weakness. However, adding another classification system risks further confusion within both clinical and research settings. A unified classification would improve diagnostic clarity, treatment planning, and research consistency.

### Treatment reporting

This review used the TIDieR checklist to extract and organise data on rehabilitation interventions, which we hope has been useful to readers. However, it also highlights the generally poor reporting of rehabilitation for NTOS. Rehabilitation descriptions are often superficial, making it difficult to replicate the methods across different settings. Excluding case studies, only two expert opinion pieces^19,38^ provide sufficiently detailed rehabilitation protocols that could be reproduced with confidence. However, these protocols remain untested. To advance NTOS rehabilitation, standardised, reproducible protocols are needed to enable comparison across studies. A dedicated study focused solely on therapy management for NTOS, designed and delivered by therapists, would significantly strengthen the evidence base.

### Recommendations

**Figure 4.**
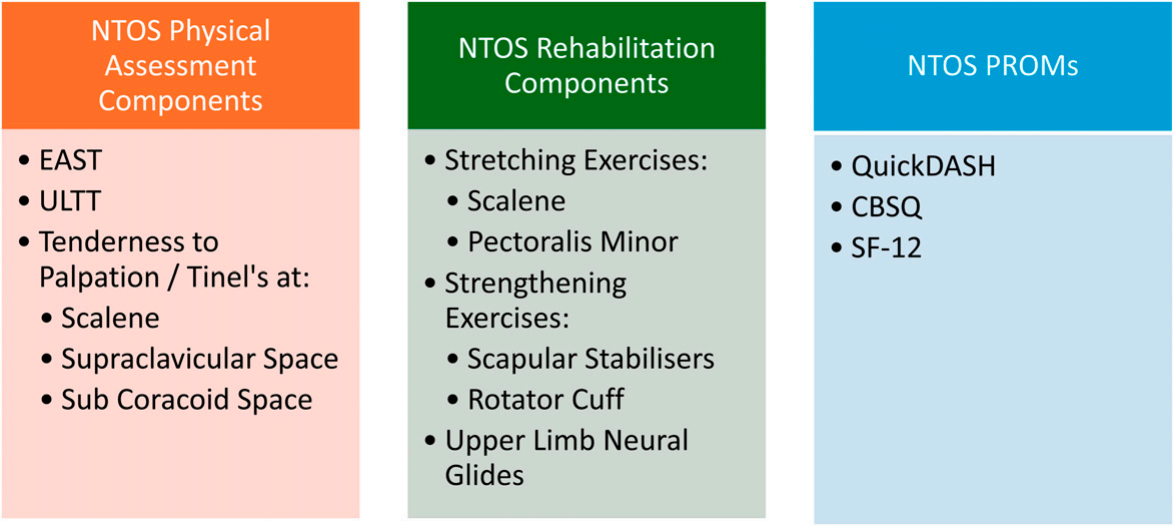
Recommendations for the physical assessment, rehabilitation and measurement of NTOS based on studies main aims and results. (CBSQ: cervical brachial symptoms questionnaire; EAST: elevated arm stress test; PROMs: patient reported outcomes measures; SF-12: short form 12; ULTT: upper limb tension test).

Based on the aims and findings of this review, we propose recommendations for physical assessment, rehabilitation, and the use of PROMs in NTOS (Figure 4).

### Limitations

This review aimed to use transparent and rigorous methods, but there are some limitations. Firstly, scoping reviews strike a balance between breadth and depth of research,^61^ and as such, there are areas we were unable to address (such as imaging and injections). Additionally, despite our efforts to identify and describe the included studies physical assessment and rehabilitation components for NTOS, it is still apparent that this remains a complex area, with no widespread conformity on the diagnosis or classification of NTOS, therefore interpretations that this article draws may not be widely generalisable. Moreover, a known limitation of scoping reviews, is the lack of quality appraisal or risk of bias assessment of included articles, therefore it is possible that studies with poor methodological quality may have led to misleading interpretations, which contrasts with a systematic review.^28^ Nevertheless, we believe this review has met its objectives and that the scoping review methodology was the most appropriate approach. Additionally, we acknowledge that some studies published before 2000 or in foreign languages may have been missed in this review.

Finally, when selecting articles based on the inclusion criteria, it was subjective to determine whether a study had described physical assessment or rehabilitation “sufficiently.” However, both reviewers (JOS & CR) discussed any disagreements until consensus was reached.

## Conclusion

NTOS is a clinically complex condition to both assess and rehabilitate for therapists working within musculoskeletal and hand therapy settings. A vast array of potential assessment and rehabilitation components for the management of NTOS are reported but are generally untested. The reporting of rehabilitation interventions for NTOS is generally insufficient, inhibiting the creation and evaluation of homogenous rehabilitation programs, which are needed to advance the evidence base concerning the therapy management of NTOS.

There is a critical need for a standardised, reproducible rehabilitation treatment regime for NTOS, ideally co-designed by both experienced NTOS clinicians and NTOS patients. The use of consistent outcomes, including both PROMs and functional markers measured pre- and post-treatment, could help establish a logic model to assess the effectiveness of rehabilitation.

## Supplemental material

Supplemental Material - Physical assessment and rehabilitation for neurogenic thoracic outlet syndrome (NTOS): A scoping review Supplemental Material for Physical assessment and rehabilitation for neurogenic thoracic outlet syndrome (NTOS): A scoping review by Joel O’Sullivan, Christian Rushton, Marcus Bateman, Caroline Miller, Claire Stapleton and Jonathan Hill in Hand Therapy

Supplemental Material - Physical assessment and rehabilitation for neurogenic thoracic outlet syndrome (NTOS): A scoping review Supplemental Material for Physical assessment and rehabilitation for neurogenic thoracic outlet syndrome (NTOS): A scoping review by Joel O’Sullivan, Christian Rushton, Marcus Bateman, Caroline Miller, Claire Stapleton and Jonathan Hill in Hand Therapy
