# Supplementary material for: Physical assessment and rehabilitation for neurogenic thoracic outlet syndrome (NTOS): A scoping review
Source: Hand Ther. 2026 Feb 5:17589983251411877. Online ahead of print. doi: 10.1177/17589983251411877 (PMC12875897; doi:10.1177/17589983251411877)
Supplement: Supplemental Material - Physical assessment and rehabilitation for neurogenic thoracic outlet syndrome (NTOS): A scoping review [file sj-pdf-1-hth-10.1177_17589983251411877.pdf]

Supporting Information 2- Physical Assessment, Measurement and Rehabilitation- Data & References

Physical Assessment

Label	Study ID	Count	Code	Specifics
Roos / EAST (Elevated Arm Stress Test)	3, 5, 7, 8, 10,11, 13, 14, 15, 17, 19, 21, 25, 26, 27, 28	16	Provocation Tests	EAST
Upper Limb Tension Test (ULTT)	5, 6, 7, 8, 10, 11, 13, 14, 15, 17, 21, 25, 26, 28	14	Provocation Tests	ULTT 'cluster better'
Palpation	5, 6, 7, 11, 13, 14, 15, 19, 25, 27, 28	11	Palpation	Pectoralis minor, scalenes, scalene triangle, subcoracoid space
Posture Assessment	11, 13, 14, 15, 21, 25, 26, 27, 28, 29	10	Posture, Observation	Postural Assessment
Adson’s	3, 10, 14, 15, 17, 19, 21, 25, 26, 28	10	Provocation Tests	Adson’s
Scapulothoracic Assessment	10, 13, 14, 15, 16, 17, 26, 27, 28, 29	10	Range of Motion (ROM), Scapula	Resting scapula position (medial border winging) /scapula dyskinesis

Supporting Information 2- Physical Assessment, Measurement and Rehabilitation- Data & References

Neurological Testing (Dermatomes and Myotomes)	3, 13, 14, 15, 17, 19, 25, 26, 28	9	Neurological Exam	Upper Limb.
Shoulder ROM	10, 13, 14, 15, 17, 25, 26, 27, 29	9	ROM, Shoulder	Not Applicable
Tinel	5, 7, 8, 13, 15, 25, 26, 27, 28	9	Tinel	Scalene triangle, Subcoracoid space
Upper Extremity ROM	3, 6, 10, 13, 14, 15, 21, 25	8	ROM	Upper Limb
Cervical ROM	11, 13, 14, 15, 17, 19, 25, 26	8	ROM	Not Applicable
Tight Pectoralis major / minor	3, 6, 14, 16, 26, 27, 28	7	Muscle Length	Pectoralis major / minor
Spurling’s	3,6, 11, 15, 25, 28	6	Provocation Tests	Spurling’s
Reflexes	13, 15, 17, 25, 28	5	Neurological Exam	Not Applicable
Tenderness on Palpation (TOP)	3, 6, 7, 11, 13	5	Palpation	Specific TOP
Muscle Length - Scalenes, Latissimus dorsi	6, 14, 16, 26	4	Muscle Length	Scalene, Latissimus Dorsi
Costoclavicular test	11, 19, 21, 28	4	Provocation Tests	Not Applicable

Supporting Information 2- Physical Assessment, Measurement and Rehabilitation- Data & References

Strength - Rotator Cuff, Scapula	14, 19, 25, 27	4	Strength Assessment	Rotator Cuff, Scapula
Manual Muscle test (MMT) - Shoulder through to Grip.	13, 14, 25	3	Neurological Exam, Strength	Not Applicable
Visual Inspection / Atrophy – Hand, Shoulder, Arm	6, 21, 25	3	Observation	Atrophy – of the upper limb
Acromioclavicular joint (ACJ), Sternoclavicular joint (SCJ), Clavicle	13, 15, 26	3	Palpation, Joint Mobilisations	ACJ, SCJ
1st Rib Mobility	15, 17, 26	3	Palpation, Joint Mobilisations	1st Rib
Anteriorly Rolled Shoulders	3, 6, 16	3	Posture, Observation	Shoulder
Postural Assessment - Neck, Thoracic Spine, Shoulder Girdle, Pelvis	6, 17, 21	3	Posture, Observation	Not Applicable
Forward Head Posture (FHP)	6, 13, 15	3	Posture, Observation	FHP
Droopy Shoulder	6, 13, 29	3	Posture, Observation	Droopy Shoulder
ROM - Active / Passive, Neck and Shoulder	6, 13, 26	3	ROM	Active / Passive, Neck and Shoulder
Reduced Grip Strength	3, 13, 14	3	Strength Assessment	Grip Strength

Supporting Information 2- Physical Assessment, Measurement and Rehabilitation- Data & References

As per 'Clinical Diagnostic Criterion' (CDC)	1, 12, 13	3	Not Applicable	Not Applicable
Cervical Spine Mobs	11, 15, 26	3	ROM, Joint Mobs	
Physical Examination	8, 28	2	Objective Assessment	Nil else detail
Cervical Kyphosis	3, 6	2	Posture, Observation	Neck
Thoracic Spine Kyphosis	13, 17	2	Posture, Observation	Thoracic
Wrights, Eden test	26, 28	2	Provocation Test	Wrights, Eden test
Allens	3, 11	2	Provocation Tests	Allens
Strength / Co-ordination	17, 19	2	Strength	Scalene, Pecs, Traps, Shoulder girdle
Rule out Cervical Radiculopathy	28	1	Differential	
Rule out golfer's elbow, tennis elbow, SIS, CTS, Cubital	15	1	Differential, Special Tests	
Muscle Length Trapezius	14	1	Muscle Length	Traps
Muscle Length-	16	1	Muscle Length	Sternocleidomastoid (SCM)
C8/T1 Fatigue	3	1	Neurological Exam	C8/T1
Cranial Nerves	3	1	Neurological Exam	Cranial
Breathing Assessment	6	1	Observation	Breathing- Diaphragmatic
Cyriax	15	1	Provocation	

Supporting Information 2- Physical Assessment, Measurement and Rehabilitation- Data & References

Manoeuvre				
Brachial Plexus Compression / Morley's	21	1	Provocation Test	
Halstead	3	1	Provocation Tests	Halstead
Allen's Pump	11	1	Provocation Tests	
Bakody's Test	11	1	Provocation Tests	
Strength- Rhomboid, Trapezius	26	1	Strength	Rhomboid

Supporting Information 2- Physical Assessment, Measurement and Rehabilitation- Data & References

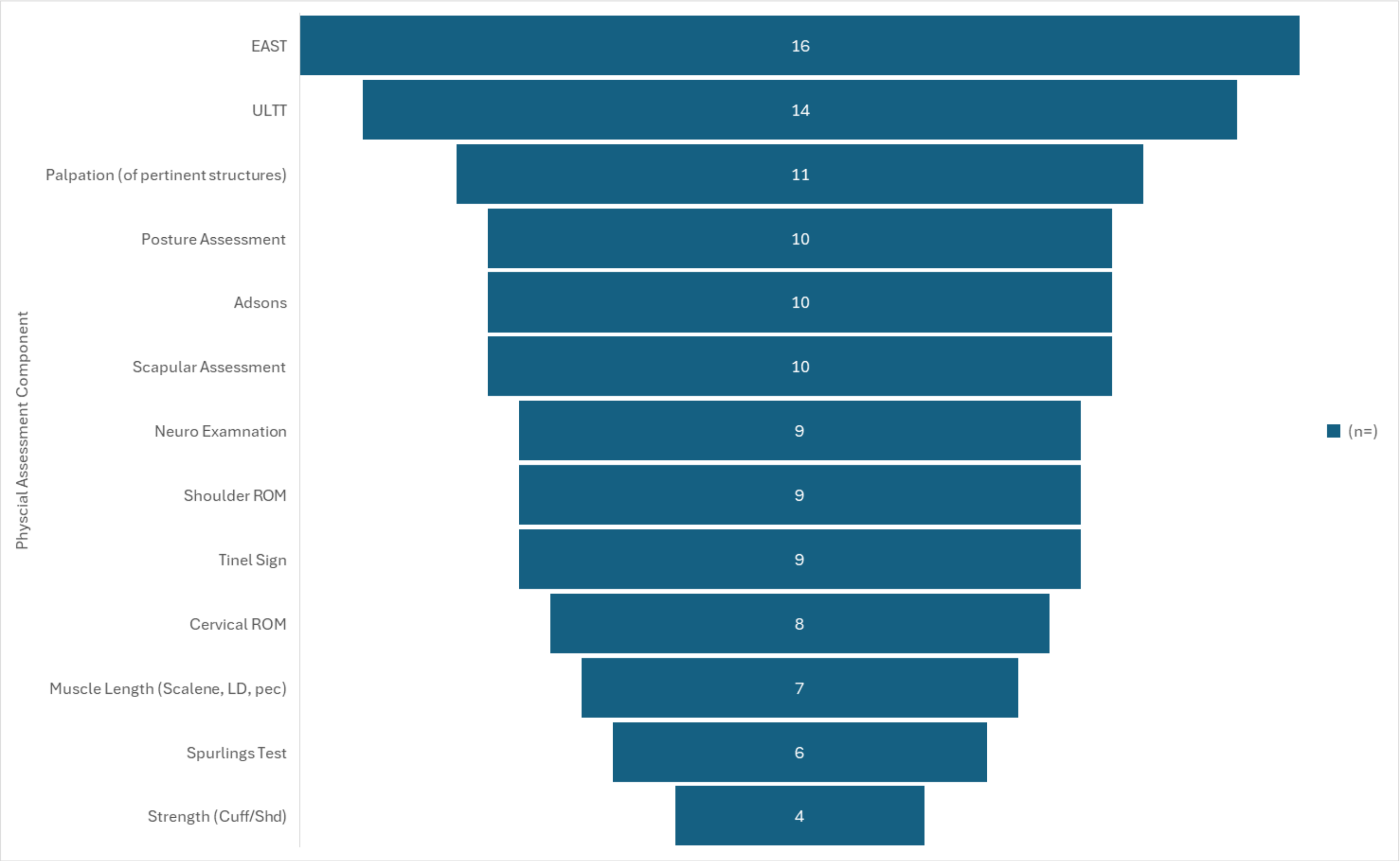

Figure 1. Funnel chart to represent most frequent physical assessment components for NTOS encountered from included studies (EAST- Elevated arm stress test; ULTT- upper limb tension test; ROM- range of motion; LD- Latissimus dorsi; Pec- pectoralis major / minor; Cuff- rotator cuff muscles; shd- shoulder).

Measurement

Supporting Information 2- Physical Assessment, Measurement and Rehabilitation- Data & References

PROMs	ID #	Count #
QuickDASH / DASH	1, 2, 5, 7, 9, 12, 13, 14, 19, 20, 22, 28	12
CBSQ	1, 2, 5, 12, 13, 14, 22, 28	8
SF-12	1, 2, 5, 12, 13, 22	6
NRS / VAS- pain	4, 5, 7, 9, 20	5
McGill Pain Questionnaire	1, 2, 17, 26	4
Brief Pain Inventory	1, 2	2
Zung Self-Rating Depression Scale	1, 2	2
Pain Catastrophising Scale	1, 2	2
NTOS Index	1, 12	2
TOS Disability Scale	12, 22	2
Northwick Park Neck Questionnaire	17, 26	2
Isokinetic Strength (grip)	7, 9	2
VAS- Paraesthesia	20	1
HandQ	5	1
Kerlan Jobe Orthopaedic Clinic	10	1
Changes in Cspine ROM	11	1
VAS for TOP Supraclavicular / Trapezius	11	1
RTP Time	19	1
Nottingham Health Profile	20	1
Degree of Improvement (Likert)	24	1

Supporting Information 2- Physical Assessment, Measurement and Rehabilitation- Data & References

Figure 2. Bar chart to display frequency of PROMs / measurement tools used / proposed to assess NTOS in included studies (RTP- Return to play; VAS- visual analogue scale; TOP- tenderness of palpation; Cspine- cervical spine; ROM- range of motion; NTOS index (composite score of QuickDASH).

Supporting Information 2- Physical Assessment, Measurement and Rehabilitation- Data & References

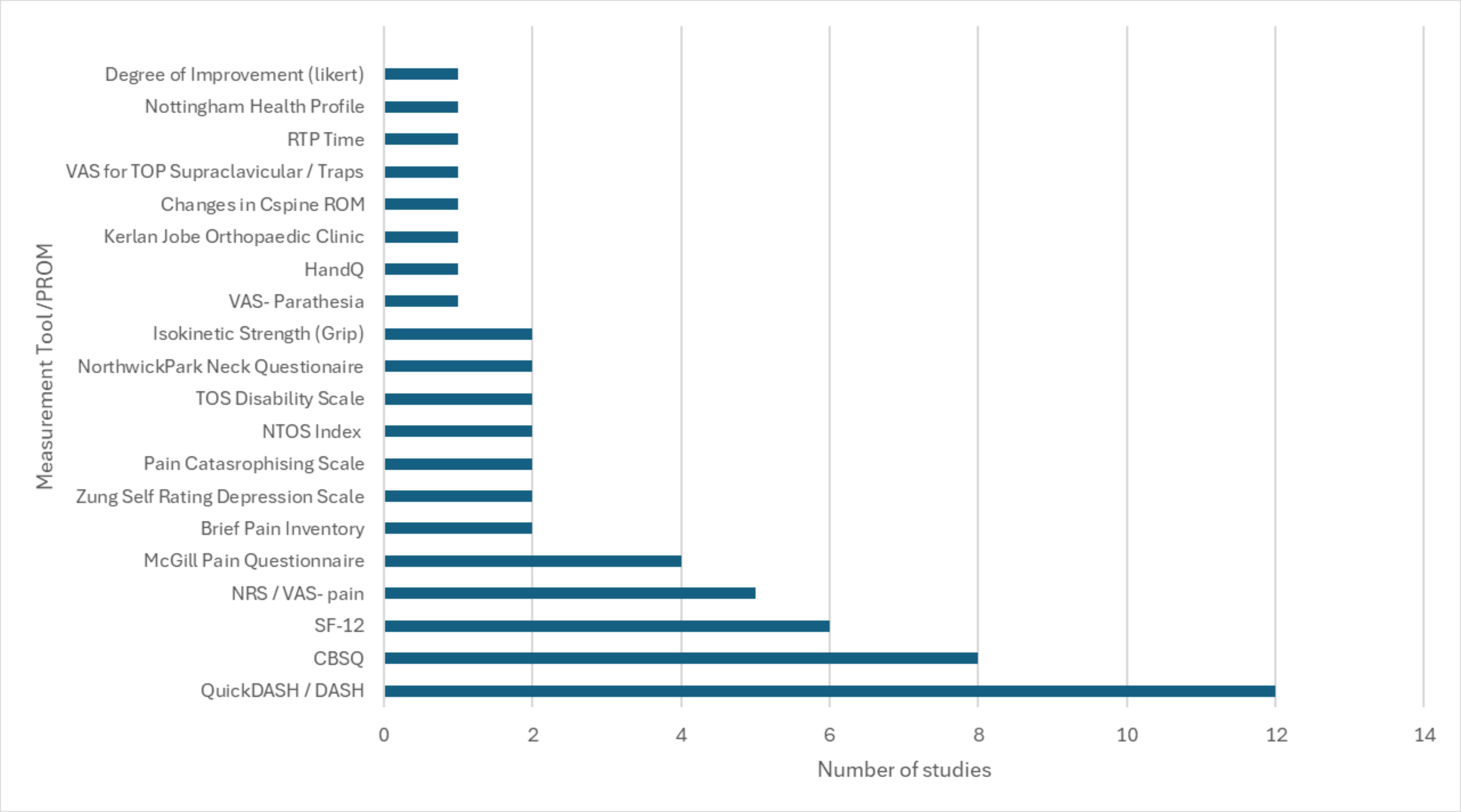

Rehabilitation- TIDieR

Tidier Criteria	Verbatim	Detail	Coded

Supporting Information 2- Physical Assessment, Measurement and Rehabilitation- Data & References

**name of intervention**	NTOS specific / dedicated physical therapy (2, 12, 19, 21)<br>Rehabilitation (3, 4, 14, 25, 29)<br>Conservative treatment (5, 17)<br>Physical therapy / physiotherapy (5, 6,12, 15, 22 'supervised' 27, 28)<br>Physiotherapeutic protocol (11)<br>HI-TOP- Hisamoto-Illig thoracic outlet: the pro-active approach (13)<br>Edgelowe (19)<br>Kinesio taping (20)<br>Hot-pack, exercise - traction (24)		Physical / physio therapy- specific to NTOS (n=19)
**why (**rationale / goal)	To improve sleep (3)<br>Aleviate stress / strain / tension of compression sites / neurovascular structures (3, 6, 14, 20, 21, 24, 28) during flexion and abduction shoulder movements and on the neck muscles (3).<br>To improve / address posture abnormalities / awareness (3, 4, 6, 10, 12, 13, 16, 22, 27, 28) via strengthening posterior thorax muscles (3, 12, 28) lengthening of shoulder girdle muscles (12, 25)<br>Upper Limb neural glides to improve NM control / mobility to the hands (3, 4, 10, 24, 28).<br>Restore width of anatomical spaces (4, 6, 14, 15) / address muscle imbalance / strength / endurance (4, 6, 22, 24, 25, 27)<br>Open the thoracic outlet (4, 14, 15, 28)<br>Better self-management (6)<br>Decrease the sensitivity of the nervous system (10, 15, 17) once this has been done can focus on Rotator Cuff strengthening (10, 16) and neuromuscular control (10)<br>Eliminate paraesthesia and to eliminate or minimise pain in back and shoulder girdle muscles (11)<br>Improve ROM and mobility (13, 14, 16, 22, 25, 28)<br>Improve / restore scapular control (14, 22, 25, 27, 28, 29)<br>Improve diaphragmatic breathing (14)		Reduce compression / stress / sensitivity of neurovascular structures (3, 6, 10, 14, 15, 17, 20, 21, 24, 28)<br>Restore width of anatomical spaces / 'open' the thoracic outlet (4, 6, 12, 14, 15, 28)<br>Improve postural abnormalities / awareness (3, 4, 6, 10, 12, 13, 16, 22, 27, 28)<br>Improve ROM, neural mobility, muscle Imbalance, strength, endurance (3, 4, 6, 10, 13, 14, 16, 22, 24, 25, 27, 28)<br>Improve / restore scapular control (14, 22, 25, 27, 28, 29)

Supporting Information 2- Physical Assessment, Measurement and Rehabilitation- Data & References

**what** (materials used- leaflets/training etc)	Advice re- activity modification / Ergonomics (3, 4, 17, 20, 24, 28, 29)<br>Patient education (6, 13, 20)<br>Posture brace (3, 14, 17, 27)<br>Exercise materials- elastic bands (3, 4, 13, 29) dumbbells (4, 13) Foam roller (3) CV equipment (6) gym ball (6)<br>Taping (6, 13, 14, 17, 20, 29)<br>Pillow / towel roll (6)<br>Heat / cold (13)<br>Instrument assisted soft tissue mob (IASTIM) (13)<br>Cupping (13)<br>Electrotherapy (TENS) (13)<br>HEP (13, 24, 29, 24, 22, 16)<br>Mirrors (29)<br>Biofeedback (29)<br>Video (29)		Advice / education- activity modification, ergonomics, posture advice (n=9)<br>Postural brace / taping (n=8)<br>Home exercise programme (n=5)<br>Exercise related e.g. resistance bands, dumbbells, foam roller, gym ball, CV equipment (n=5)<br>Mirrors, video, biofeedback (n=1)<br>Heat / cold / IASSTIM /electrotherapy (n=1)
**what** (procedures, activities)	Exercise (n=17)<br>Stretching / Mobility / Elongation (n=15)<br>Strengthening (n=14)<br>Neural Mobility (n=7)<br>Diaphragmatic breathing (n=6)<br>Range of motion (n=3)<br>Cardiovascular (n=1)<br>Posture improvement (n=13)<br>Via exercise (n=11)<br>Brace (n=1)<br>Via education (n=1)<br>Manual Therapy (n=10)<br>Soft tissue massage (n=7)<br>Joint mobilisations (n=5)<br>Therapist led passive stretches (n=3)<br>Trigger point therapy (n=2)<br>Manipulations, cupping, dry needling, traction (n=1)<br>Adjuncts (n=8)<br>Taping (n=6)		

	Posture brace (n=4)<br>Activity Modification (n=7)<br>Inhibit ant chest / stop OH activity / avoid shoulder drooping / sleep advice (n=1)<br>Psychosocial informed treatment (n=1)<br>CBT / motivational interviewing / self-efficacy / adherence / understanding / meditation – relaxation (n=1)		
**who** provided	Physical therapists (2, 6, 11, 13, 14, 15, 16, 24, 27, 29)<br>Athletic trainer / team physician (3)<br>Specialist TOS physio (4, 12, 22)<br>Not specified (5,)<br>Patient / self (16, 29)<br>Certified physician (20)<br>'PT' that works with varsity athletes (19)		Physio (n=14)<br>Physician (n=2)<br>Self (n=2)<br>? (n=1)
**how** provided	Face to face (3, 4, 11, 13, 16, 20, 22, 24, 27, 28, 29)<br>Not stated (2, 5, 6, 12, 14, 15, 17, 21)		F2F / supervised (n=11)
**where**	Physio clinic (2, 3, 4, 11, 12, 13, 16, 20, 22, 24, 29<br>Home (16, 24, 29<br>Not stated (6, 14, 15, 17, 21, 27, 28		Physio clinic (n=11)<br>Physio clinic plus home (n=3)
**when & how much** (dose, frequency, duration)	When-<br>Majority of more than 2 years (1, 2, 7, 9, 22)<br>10.7 months (24)<br>7.1 months (10)<br><3 months 64% (19)<br>at least 3 months (20)<br><br>Case study- less than a week (3)	6 weeks (2, 15)<br>6 weeks for acute (mild) pts- 12 weeks for chronic (severe) (13)<br>3 weeks- 8 sessions (11)<br>4-6 months (17)<br>3 weeks to resolution (3)<br>3-6 months (5)<br>min 6 months- otherwise not described (4)<br>6 months (16, 21, 27)	**When**<br>Most interventions (n=5) occurred when the majority of participants had symptoms present for more than 2 years (1, 2, 7, 9, 22)<br>3 studies (24, 10, 20) participants had symptoms for at least 3 months<br>2 studies less than 3 months (19, 3) with a case study involving a patient with symptoms lasting less than a week (3)<br><br>**Intervention length**<br>6 months was the most commonly reported

		min 6 weeks, max of 12 weeks (22, 29)<br>12 days (20)	timeframe suggested for therapy (n=6), all were review / consensus articles except one retrospective analysis by Camporese et al (4)<br>3 months of continued therapy for patients previously refractory to improvement with therapy was delivered in an RCT (12)<br>6-12 Weeks was proposed by two expert opinion articles (13, 29) and delivered in one prospective observational study of 476 NTOS patients (22)<br>Balderman et al (2019) (2)- provided 6 weeks of therapy to 150 NTOS patients in a prospective observational study, and was proposed by another review article (15)<br>two case reports, both reported treatment lasting 3 weeks (3, 11), and RCT (24) involving cervical traction lasting for 2 weeks<br>an RCT involving Kinesio taping lasted for 12 days (20)<br><br>**Frequency / session duration**<br>Nil relevant information on dosage in ten articles (2, 4, 5, 6, 12, 15, 16, 17, 27, 28)<br>RCT (24) delivered 10 sessions, 5 days a week for 35-45 mins of hot packs and cervical traction- 20 seconds on- 10 seconds off with 10-15kg of traction weight<br>another RCT (20) provided 3 separate sessions of Kinesio taping for 4 days each<br>A prospective observational study, recommended daily unsupervised exercise, and 1 session per week of supervised input (22)

			2 studies provided dosage information for stretching exercises (3, 13)- one stating 4 sets of 30 second holds, the other 20 second holds, for 3-5 repetitions (13)<br>for strengthening exercises- 2-3 sets of 8-15 repetitions were recommended (3). 4 articles (4, 13, 14, 28) advised a 'high reps, low weight' approach when performing strengthening exercises to begin with<br>for isometric / postural setting exercises, Hisamoto (13) recommended 5 second holds, for 5 repetitions for 2 sets each day<br>Nerve gliding exercises were recommended with caution to begin with, progressing up to 25 repetitions up to 2-5 times daily (13)<br>Watson (29) recommended a dosage of 20 reps x3 sets per day for all type of exercises related to NTOS<br>Cardiovascular exercise of 30 minutes pr day was recommended by 1 study (6)<br>2 expert opinion articles, recommended a staged approach to exercise progression (13, 29)
**tailoring** any modifications	Scapular based exercises added if scapula dyskinesis noted (3)<br>Focus on controlling symptoms in high SIN (6)<br>Dependent on SIN (14)<br>Adjust weight of traction if needed (24)<br>Short lever / long lever (29)<br>Use of pain relief, injections, TENS, Ultrasound or thermal agents if helps with symptoms (28)		Can be tailored depending on patients SIN factor (6, 14, 28), addition of TENS, pain relief, injections, ultrasound and thermal agents to manage symptoms if helpful (28)<br>Addition of scapular based exercises if scapula dyskinesis is noted (3)<br>Use of short or long lever exercises based on progress (29)

			Weight of traction can be adjusted if needed (24)
**modifications**	n/a		
**how well planned** (intervention adherence / fidelity)	Achieved full follow up data for 21.1 months (2)<br>Full follow up of 285 patients for 6/12 (4)<br>Continued physiotherapy patients were dissatisfied with treatment and crossed over to surgical treatment (12)<br>full follow up (12, 20, 24)<br>Can expect a reliable change in resting scapular position in 6 weeks and strength / scapular motion in 12 weeks (29)		Full follow up (n=5 2, 4, 12, 20, 24)<br>Dissatisfied with physio so opted for surgery (12)<br>Could expect changes in scapular posture in 6 weeks, and strength in 12 weeks if adhered to exercise programme (29)

Exercise

Supporting Information 2- Physical Assessment, Measurement and Rehabilitation- Data & References

Category	Intervention Components	Target	Detail
**Exercise**<br>(2, 3,4 5,6, 11,12, 13, 14, 16, 17, 21, 22, 24, 27, 28, 29)	**Stretching / Mobility**<br>(2,3,6, 11,12, 13, 14, 16, 22, 24, 27, 28, 29)<br>**Elongation exercise** (5, 17)<br>**Range of Motion** (12, 16)<br>**Tendon gliding exercises** (21)	Scalene<br>(2, 5, 6, 11,12, 13-stage 2, 16, 24, 28, 29)<br>Pectorals<br>(2, 6,12, 13-stage 2, 14, 16, 24, 27, 28, 29)<br>Scapula (2, 22)<br>Shoulder girdle (2, 22)<br>Anterior thorax (3)<br>Lat dorsi (6)<br>Levator Scapulae (11,12, 13-stage 2, 28)<br>Traps<br>(11, 13-stage 2, 28)<br>SCM (11)<br>No further details (17)<br>--------------<br>Cervical spine (12, 17)<br>Shoulder (16, 17)<br>-----<br>No further details (21)	As per 'Edgelowe' (2)<br>Super 6 foam roller exercise (3)<br>Crucifixion stretch (3)<br>Child's pose (3)<br>Pec stretch in supine if irritable +/- contract-relax (14)<br>Pec doorway / Ball stretch (6)<br>-----------<br>Start 0-30<br>Progress 45-90 then overhead in scaption plane
	**Strengthening**<br>(3, 4, 5, 6, 12, 13, 14, 16, 17, 21, 24, 27, 28, 29)	Post thorax muscles(3)<br>Inferior scapular muscles (12)<br>Periscapular muscles (21)	Banded I's, T's Y's (3)<br>Upright rows (3, 4)<br>Serratus punches (3, 4)<br>Scapular

		----------------------------<br>---<br><br>Core stability (3, 6,<br>14)<br>----------------------------<br>-<br>Rhomboids (4, 16,<br>28)<br>Mid-lower trapezius<br>(4, 13- stage 2, 14,<br>16, 28)<br>Supraspinatus (4,<br>13-stage 2, 4)<br>Infraspinatus (4, 13-<br>stage 2, 4)<br>Deltoid (4)<br>Lat dorsi (4)<br>Teres major & minor<br>(4)<br>Serratus anterior (4,<br>113-stage 4, 14, 16,<br>28)<br>Triceps (4)<br>Rotator cuff (13-<br>stage 2, 3, 4)<br>Shoulder elevation<br>(13-stage 2)<br>Neck erectors (16)<br>Scalene (21)<br>Pec minor (21)<br>Pectoral (24)<br>Shoulder elevator<br>(24)	retractions (3, 4, 5,<br>13-stage 1)<br>Scaption on a wall<br>(3)<br>Scapular clocks (3)<br>Cuff isometrics<br>(13- stage 3)<br>start isometric (14)<br>-------------------------<br>---<br>Dead bugs (3)<br>Bird dogs (3)<br>No further detail<br>(14)<br>-------------------------<br>--<br>Standing /prone<br>Shoulder ext,<br>horizontal Abd (4,<br>29)<br>Scapula<br>depressions (4, 13-<br>stage 1)<br>Front raises (4)<br>Standing shd ER<br>(4)<br>Shd lateral raises<br>(4)<br>Straight arm ext.<br>(4, 29)<br>Chin tucks (4, 13-<br>stage 1)<br>Resisted chin<br>tucks (13-stage 2)<br>Dynamic cuff in

		----------------------------<br>-<br><br>Scapular Stabilisation (5, 6, 12, 13- stage 1,2,3, 4, 14, 27, 28, 29- stage 1, 2, 3, 4, 5)<br><br>-------------------------<br><br>CV exercise (6, 13- stage 3)<br><br>--------------------<br>Distal kinetic chain with oscillations (13- stage 4)<br>-------------------<br><br>Recruitment of stabilisation-based muscle and inhibition of over-utilised muscles (15)<br><br>No further details (17)	scaption (13-stage 4)<br>Dynamic serratus ant in scaption (13- stage 4)<br>No further detail (16, 21, 24)<br>Upward rotation shrug (29)<br>Upward rotation shrug with ER side lye (29)<br><br>-------------------------<br>---<br>Scapular stabilisation & retraction (5)<br>Scap stabilisation- avoid retraction/depressi on (6)<br>At rest, then passive, then active through whole range (12)<br>Brugger scapular stab / ULNTT2 (13- stage 2)<br>Scap stabilisation- Eccentric bias- short/long lever (13- stage 3)<br>Scap setting with cuff- progress into

			elevation (13-stage 4)<br>Scap kinematics- No further detail (27)<br>Scap setting (29-stage 1)<br>Scap setting <30- upward rotation shrug (29-stage 2)<br>Scap control 45-90 of Abd (29- stage 3)<br>Scap control in flexion (29- stage 4)<br>Scap /Humeral Head control >90 with resistance (29- stage 5)<br><br>-----------------------<br>No further details (6, 13-stage 3)<br>-----------------<br>No further details (13- stage 4)<br>------------------------<br>No further details (15)
	**Cardiovascular** (6)		
	**Neural mobility** (3, 5, 6, 13, 14, 21, 28)	Upper limb neural glides (3, 5, 6, 14, 21, 28)	No further detail (3,5,6, 21, 28)<br>Brugger scapular

		Scap stab / ULNT2 (13-stage 2)<br>ULTT 1-4 (13, stage-2)	stabilisation/ ULNTT2 (13-stage 2)<br>Start gentle (13-stage 2)<br>Median, Ulnar-pain free (14)
	**Diaphragmatic breathing** (2, 4, 6, 13, 14, 24)	No further detail (2,4,6,13, 14, 24)	
**Posture improvement** (2,3,4, 5, 6,13, 15, 20, 21, 22, 24, 27, 28)	**Via exercise** (2,3,4, 5, 6,13,15, 21, 22, 24, 28)<br>**Via brace** (3)<br>**Verbal education** (20)<br>**No further detail** (27)	No further detail (2,3,4,5, 15, 21, 22, 24)<br>Shoulder girdle elevation / scapular protraction (6)<br>Correct dysfunctional patterns (upper crossed syndrome) (13-stage 1)<br>Reduction of FHP & Scap protraction (28)	a) Chin tuck (13-stage 1)<br>b) Scapular depression & adduction (13-stage 1)<br>c) Chin tuck with scapular depression & adduction-(13-stage 1)
**Manual Therapy** (3, 4, 5, 6, 11, 13, 14 15, 17, 24)	**Soft tissue massage** (3, 6, 11, 13-stage 3, 15, 17, 29)<br>**Therapist led passive stretches** (4, 6, 13-stage 2)<br>**Joint mobilisations** (4, 6, 13 - stage 3, 14, 29)<br>**Trigger point therapy** (5, 11)<br>**Joint manipulations** (11)<br>**Cupping** (13-stage 3)<br>**Dry needling** (14)<br>**No further details** (14)<br>**Traction** (24)	Pectorals (3)<br>SCM (3,11)<br>Scalene (3, 11)<br>Upper traps (3, 11)<br>No further detail (6, 13- stage 3, 15, 17, 29)<br>Levator scapulae (11)<br>----------------------<br>Scalene (4, 6)<br>Subclavius (4)<br>Pec minor (4,6, 13-stage 2)	Posterior capsule of shoulder (14)

		Trapezius (4)<br>-----------------<br>Cervical spine (4,6, 29)<br>1st rib (4, 6, 13-stage 3, 14, 29)<br>Ribcage (6)<br>Shoulder (6, 14)<br>Thoracic spine (14, 29)<br>Cervicothoracic spine (14)<br>-------------------<br>No further detail (5)<br>Trapezius (11)<br>SCM (11)<br>--------------------<br>rotational manipulation to C2-C6 (11)<br>------------------<br>No further details (13-stage 3)<br>-------<br>Cervical traction (14)	
**Therapy adjuncts** (3, 6, 13, 14, 17, 20, 27, 29)	**Posture brace** (3, 17, 27, 29)<br>**Taping** (6, 13, 14, 17, 20, 29)<br>**Acupuncture** (17)	Taping into shoulder / scapular elevation (6)<br>No further detail (13, 17, 27)<br>To facilitate scapular upward rotation / elevation 'axillary sling' (14, 29)	

		Kinesio taping- 4 strips (scalene, pec min, biceps, subclavian) (20	
**Activity Modification** (2,4,5, 6, 12, 24, 28)	**Cautions with weights / strengthening** (2)<br>**Cessation of overhead activities** (4)<br>**Inhibit anterior (chest / neck) pressing exercises** (4, 6)<br>**Avoid shoulder 'drooping' activities** (5)<br>**Sleep position advice** (6)<br>**Deep manipulation of 1st rib not performed due to risk of aggravating symptoms** (12)<br>**Avoidance of irritating positions** (24)<br>**No further info** (28)		
**Psychosocial informed** (6)	**Cognitive Behavioural Therapy** (6)<br>**Motivational interviewing** (6)<br>**Self efficacy** (6)<br>**Importance of exercise adherence** (6)<br>**Patient understanding** (6)<br>**Meditation / relaxation** (6)		

Figure 3. A list to represent further details of the main rehabilitation elements found within the included studies. Body parts / muscles / exercise type targeted by each element are provided along with their frequency (n=). Article referencing is available in Supplementary Information 2.

Supporting Information 2- Physical Assessment, Measurement and Rehabilitation- Data & References

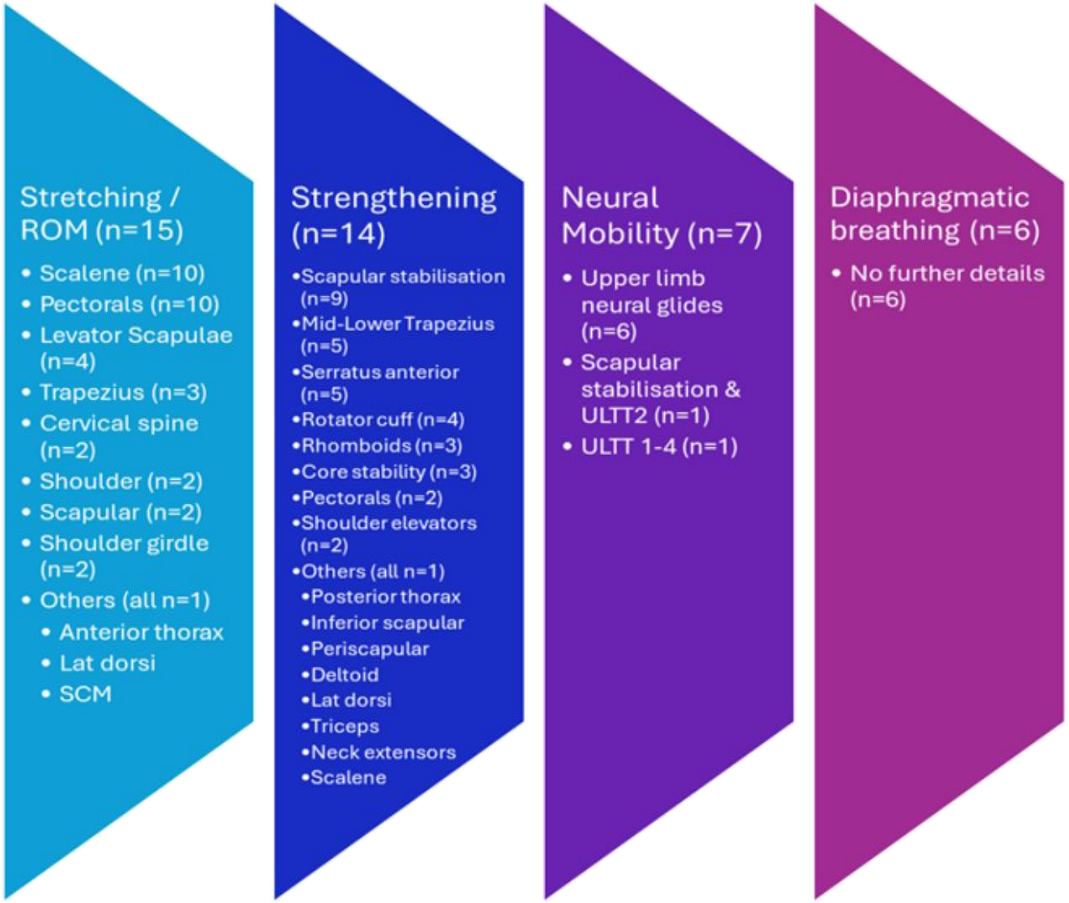

Subclassification of NTOS (Adapted from Rochkind et al, 2023)

Supporting Information 2- Physical Assessment, Measurement and Rehabilitation- Data & References

**Type of NTOS**	**Weakness and / or atrophy**	**Anatomic Abnormality**	**Pain & Sensory Symptoms**
**NTOS 1**	Yes	Yes / No	Yes / No
**NTOS 2**	No	Yes	Yes
**NTOS 3**	No	No	- Radicular **(NTOS 3a)**<br>- Cervicoscapular **(NTOS 3b)**<br>- Diffuse **(NTOS 3c)**

References (see main document for full reference)

Study	Study Reference & Country of

Supporting Information 2- Physical Assessment, Measurement and Rehabilitation- Data & References

ID	Origin
1	Balderman (2017)<br>USA
2	Balderman (2019)<br>USA
3	Berardo et al. (2024)<br>USA
4	Camporese (2022)<br>Italy
5	Chim (2024)<br>USA
6	Collins & Orpin (2021)<br>USA
7	Daley (2021)<br>France
8	Dengler (2022)<br>Germany
9	Fouasson-chailloux (2022)<br>France
10	Garrison (2021)<br>USA
11	Glowa & Trybulec (2024)<br>Poland
12	Goeteyn (2022)<br>Netherlands
13	Hisamoto (2021)<br>USA
14	Hock (2024)<br>USA
15	Kuwayama (2017)<br>USA
16	Levine (2018)<br>USA
17	Li (2021)

	USA
**18**	Luu (2022)<br>Canada
**19**	Olson (2023)<br>USA
**20**	Ortac (2020)<br>Turkey
**21**	Panther (2022)<br>USA
**22**	Pesser (2021)<br>Netherlands
**23**	Rochkind (2022)<br>Israel
**24**	Taskaynatan (2007)<br>Turkey
**25**	Troyer (2021)<br>USA
**26**	Vanti (2007)<br>Italy
**27**	Wagner (2023)<br>USA
**28**	Warrick (2021)<br>USA
**29**	Watson (2010)<br>Australia
